# Assessing the deployment of solar-driven hydrogen from biomass at scale in the U.S.

**DOI:** 10.1038/s41598-025-90290-y

**Published:** 2025-04-24

**Authors:** Chukwunwike O. Iloeje, Sarah Runchey, Audrey Gallier, Doris Oke, Li Yu, Alinson Santos Xavier

**Affiliations:** Energy Systems and Infrastructure Analysis Division, Argonne National Laboratory, Lemont, IL 60439 USA

**Keywords:** Renewable energy, Bioenergy, Hydrogen energy

## Abstract

Solar hydrogen from biomass gasification is a promising technology to sustainably produce hydrogen, responsibly dispose biomass waste, and reduce reliance on fossil fuels. However, its large-scale deployment faces challenges due to the geospatial misalignment between biomass resources and solar intensity, which introduces additional supply chain logistics costs. We analyze the logistics cost burden imposed by this misalignment and its impact on successful large-scale deployment of solar-driven hydrogen from biomass in the United States. We also consider associated carbon emissions and explore how the mix of deployed technologies evolves under externally imposed carbon penalties. Our findings show that while economies of scale are known to apply at the processing facility level, the reverse effect occurs at the broader systems-level, driven by logistics. Also, at current technology costs, high carbon penalties would be required to favor deployment of solar based technologies over conventional and hybrid alternatives. We further illustrate strategies and system-level changes to reduce logistics costs and enable sustainable, low-cost hydrogen for decarbonizing different industrial sectors.

**Supplementary Information:**

The online version contains supplementary material available at 10.1038/s41598-025-90290-y.

## Introduction

**Fig. 1:**
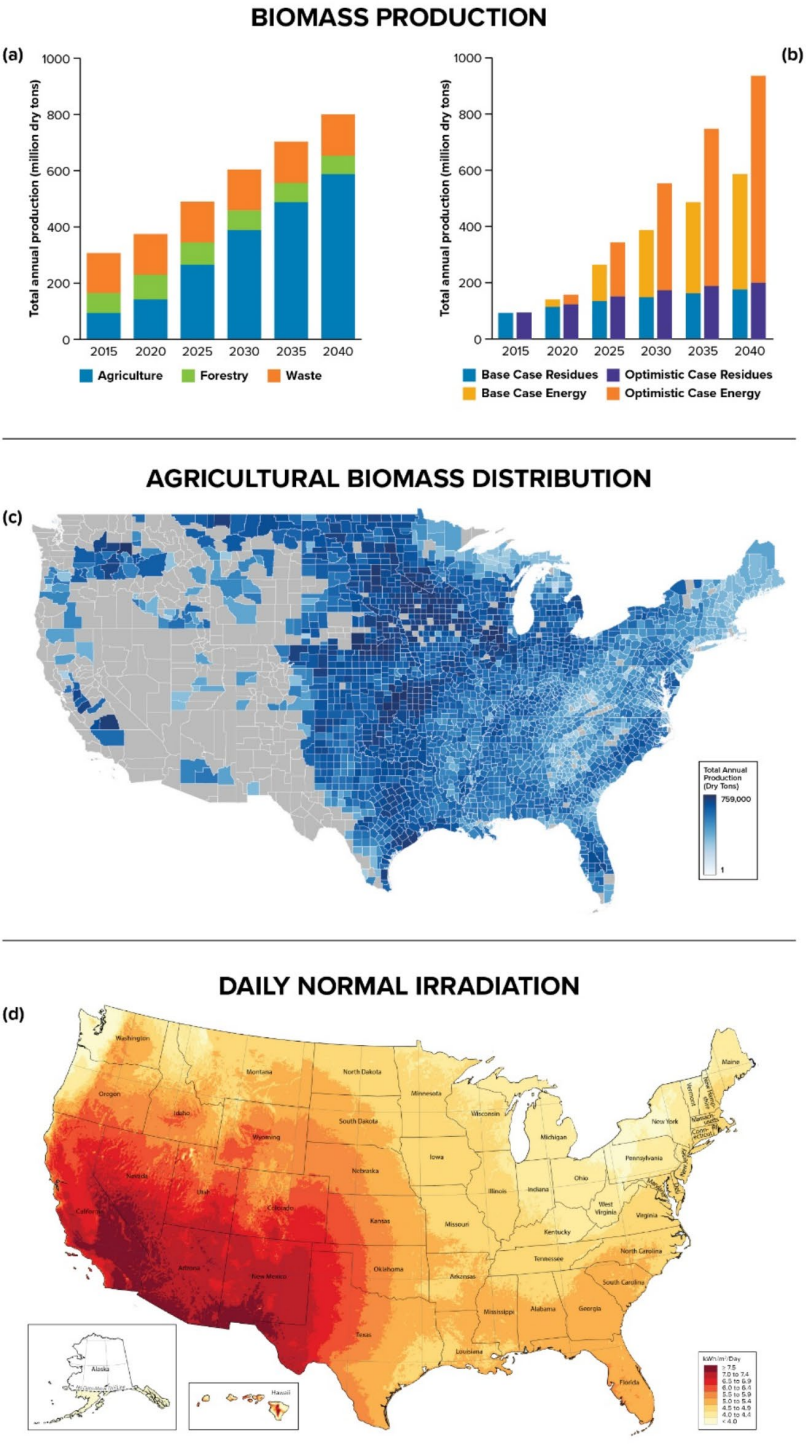
Biomass residue availability projections, biomass resource distribution, and U.S solar resource distribution data (data included with “Supplementary Material”). (**a**) Biomass availability projections, 2017–2040. (**b**) Agricultural biomass availability projections, 2015–2040, showing base case and optimistic scenarios; the current study uses the base case projections. (**c**) Map of annual agricultural biomass resource distribution in the U.S.^8^ (**d**) Map of U.S. daily normal solar irradiation data.

Sustainable production of hydrogen at the scale required to support the transition to a net-zero global economy calls for accelerated breakthroughs of more abundant, affordable, and reliable clean hydrogen solutions. However, such large-scale production faces critical barriers associated with the hydrogen value chain, especially the high production cost, as well as limited cost-competitive infrastructure for both feedstock and product distribution. To motivate a significant cost reduction, the U.S. Department of Energy (DOE) recently announced the hydrogen Earthshot target, aiming to lower the price of clean hydrogen to $1000 per tonne by 2030^1^. This target, similar to others across Europe^2^ and Asia^3^, represents a 30–50% average reduction over state-of the art steam methane reforming technology^4^, and 80–85% reduction over current costs for hydrogen from water electrolysis^5^. The Earthshot targets a cost-level that will overcome the barriers to market entry and unlock the market competitiveness for hydrogen as a primary clean energy vector across U.S. industrial and other economy sectors. These entry threshold prices for hydrogen range from $4–$5/kg for commercial trucks and buses, to $1–$2$/kg for grid storage applications^5–7^. Achieving this target will reduce the investment risk for developing and expanding the deployment of hydrogen technologies, especially for hard-to-decarbonize sectors with limited feasible alternatives. Such de-risking is critical to enabling large scale hydrogen production. Biomass residues hold significant potential to meet projected hydrogen demand and enable net-zero hydrogen production. Annual biomass residue in the US is projected to exceed 800 million tonnes by 2040, up from 330 million in 2017^8^ (Fig. 1a), and 1–1.5 billion by 2050^9^. This number includes the three main categories of biomass resources: *waste biomass*, such as municipal solid waste, secondary crop residues, and animal manure; *forest biomass*, such as whole trees and residues like small branches, woodchips, and sawdust; and *agricultural biomass*, such as dedicated energy crops like switch grass and energy cane, as well as crop residues like corn stover and wheat straw^10^. Although agricultural residues are currently the least utilized type of biomass resource, they are essential for large-scale bio-derived hydrogen production in the United States, given that they are expected to account for over 75% of the projected biomass growth^10^, a 400% increase over the next two decades. This growth will be largely driven by increased demand for dedicated energy crops, as well as improvements in sustainable production techniques for these crops (Fig. 1b). To grasp the potential scale, if all the available agricultural biomass in the U.S. is converted to hydrogen at 70% efficiency^11^, the U.S. could produce over 40 million tonnes of hydrogen annually by 2040, nearing the projected demand of up to 50 million tonnes by 2050^5,12^. Unfortunately, very little of this potential is realized as more than half of the biomass produced annually is incinerated or left in open fields to rot, a hazardous practice that can also cause or exacerbate wildfires, not to mention the $12 billion/year loss in potential market value^13^.

**Fig. 2:**
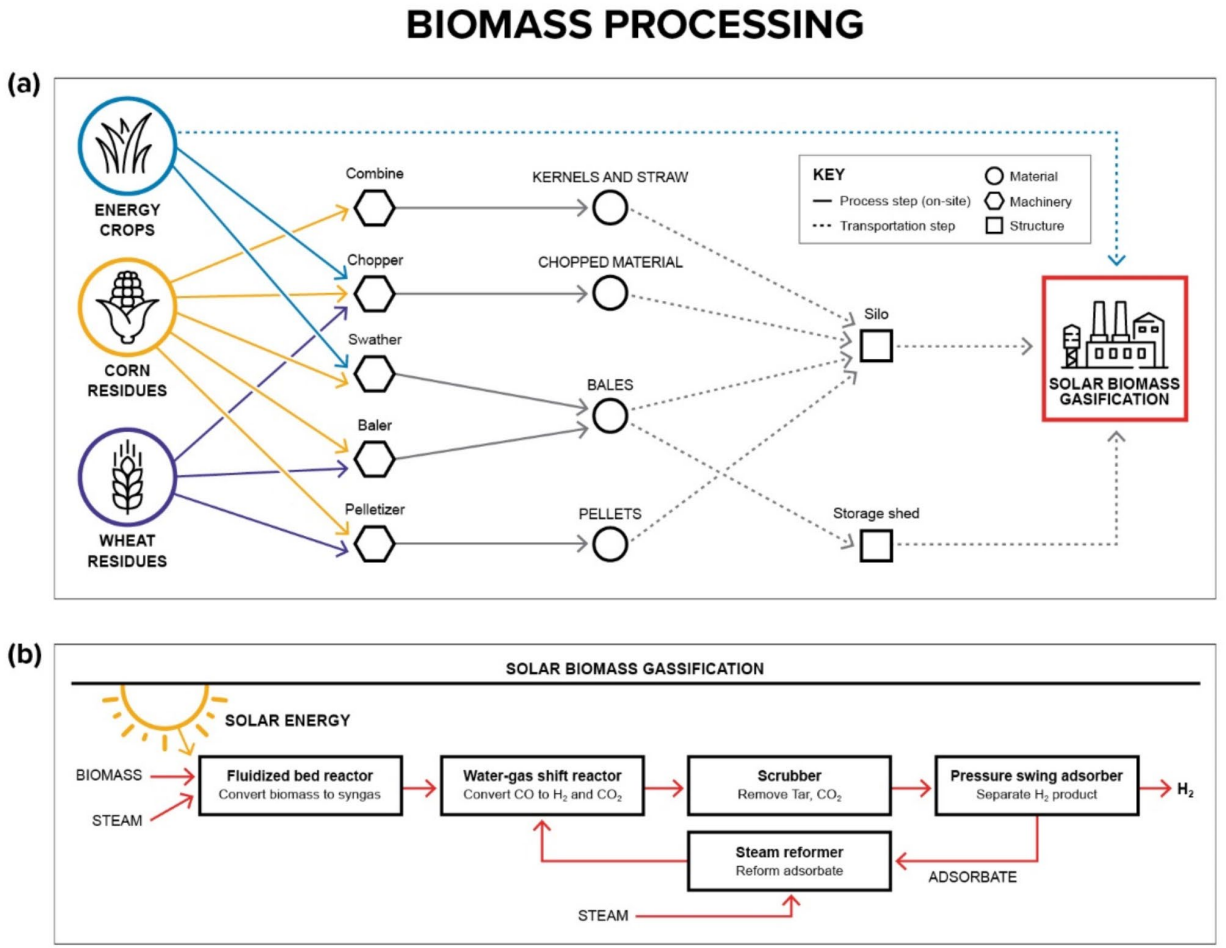
Overview of hydrogen production supply chain from agricultural biomass. (**a**) Biomass supply pathways from agricultural residue to hydrogen production plant. (**b**) High-level process flow of a solar hydrogen from biomass gasification plant^11^.

The pathway for hydrogen production from biomass starts with feedstock harvesting^8,14^. After harvesting, the residues are dried passively or actively, and subsequently processed, following typical pathways illustrated in Fig. 2a. Conversion to hydrogen happens primarily via gasification—typically in a dual fluidized bed system—accompanied by gas cleanup and hydrogen separation^11^, as illustrated in Fig. 2b. The dual fluidized bed consists of a gasification reactor and combustion chamber. Circulating bed material carries heat from the combustion reactor to the gasification chamber, and any solid waste products produced from gasifying biomass are recycled back into the combustion chamber, minimizing waste.

Switching the heat source to solar energy creates the possibility for net-zero or carbon-negative hydrogen production (Fig. 2b). For this solar hydrogen from biomass gasification (SHBG) system, concentrated solar power replaces the combustion reactor.^15^ Here, the traditional direct gasifiers are replaced by solar collector reactors—such as cavity-type reactors with packed or circulating bed material—designed with large internal areas and focused apertures for concentrated solar radiation^16–19^. Solar tower configurations^20,21^ can be used to concentrate solar radiation from a heliostat field to achieve the target temperatures required for gasification. By avoiding the consumption of biomass to drive the gasification process, this approach increases hydrogen yield compared to conventional gasification.

A number of studies have looked at solar hydrogen from biomass gasification at the facility level, focusing on strategies to improve yield and optimize production cost. Boujjat et al.^22,23^ discussed the performance and techno-economics of solar hydrogen from biomass, comparing solar, conventional and hybrid pathways—which switches from solar to biomass combustion to provide heat when solar energy drops at night. Xu et al.^24^ explored solar-driven chemical looping biomass gasification which creates the option of producing separate hydrogen and syngas streams, allowing for flexible adjustment of the carbon-to-hydrogen ratio. Others studies by Deshmukh et al. and Xin et al. evaluated solar thermochemical^25^ and hybrid thermo-electrochemical^26^ technologies for optimizing hydrogen yield and improving both energy conversion and carbon efficiency. At the level of the supply chain, several studies focused on optimizing biomass supply chains in general to enhance the efficiency of biofuel production without particular preference for hydrogen production or the use of solar. Sahoo et al.^27^ developed a GIS-based discrete event simulation model to estimate feedstock flow rate and delivery costs for a Miscanthus biomass supply chain in Ohio. Sokhansanj et al.^28,29^ outlined the logistics of lignocellulosic biomass supply chains, identifying key unit operations such as collection, storage, preprocessing, and transportation as major challenges for biorefineries and highlighting computational advances in optimizing integrated biomass systems. A number of studies have also looked at analysis and tools for regional-scale solar-driven hydrogen production from biomass in other countries, with focus ranging from site selection to technoeconomics^30–32^. More recently, Reed et al.^33^ reported a study on the build-out of new renewable hydrogen facilities to meet the projected demand for California, considering a range of technologies from steam methane reforming (SMR) to thermochemical biomass conversion and highlighting the need for rapid infrastructure expansion. While these studies provide valuable frameworks and insights directly or indirectly relevant to assessing the feasibility of hydrogen production from biomass gasification, they tend to focus on individual facilities or limited sub-regional supply chains that do not capture the effect of misaligned distribution of solar and biomass resources, with scales too small to assess the practical limits of meeting projected hydrogen demand.

Yet, the main challenge with using solar power for biomass gasification at scale is the misalignment between the geospatial distribution of agricultural biomass resources and solar irradiation intensity across the United States, as illustrated in Fig. 1c,d. Solar irradiation is highest west of the Rocky Mountains, while agricultural biomass is mostly found to the east. This misalignment can create significant supply logistics challenges and disproportionately high transport costs as the feedstock collection radius increases. Consequently, the cost of hydrogen could increase in proportion to the production scale, adding to the economic barrier to affordable solar hydrogen from biomass at scale. Therefore, assessing the true potential of *biomass to hydrogen* requires consideration for the complex interplay and trade-offs between logistics and the conversion technology, in the context of their associated economics and carbon emissions. Our key contribution to this space is the assessment and quantification of supply chain features and trends that emerge at very large scales, such as those needed to meet 100% of projected U.S. hydrogen demand up to 2050. We uniquely explore the interplay and implications of biomass and solar resource misalignment on supply logistics and costs, enabling the quantitative evaluation of the potential and practical limits of meeting hydrogen demand in this context.

In this study, we investigate the techno-economics of large-scale deployment of hydrogen from biomass gasification technology, highlighting the impact of scale on hydrogen production cost, given the misalignment in solar and biomass resource distribution in the U.S., and its implications for biomass feedstock logistics. We identify the major cost drivers and how they evolve with scale, and also assess the impact of carbon penalties on the evolution of deployed biomass-to-hydrogen technology mix when considering alternative biomass gasification pathways. We present study outcomes in the context of the economics of competing hydrogen production technologies and assess the impact of strategies and system-level changes that can make hydrogen production cost-competitive for decarbonizing end-use industrial sectors.

## Results

### The economic impact of logistics

**Fig. 3:**
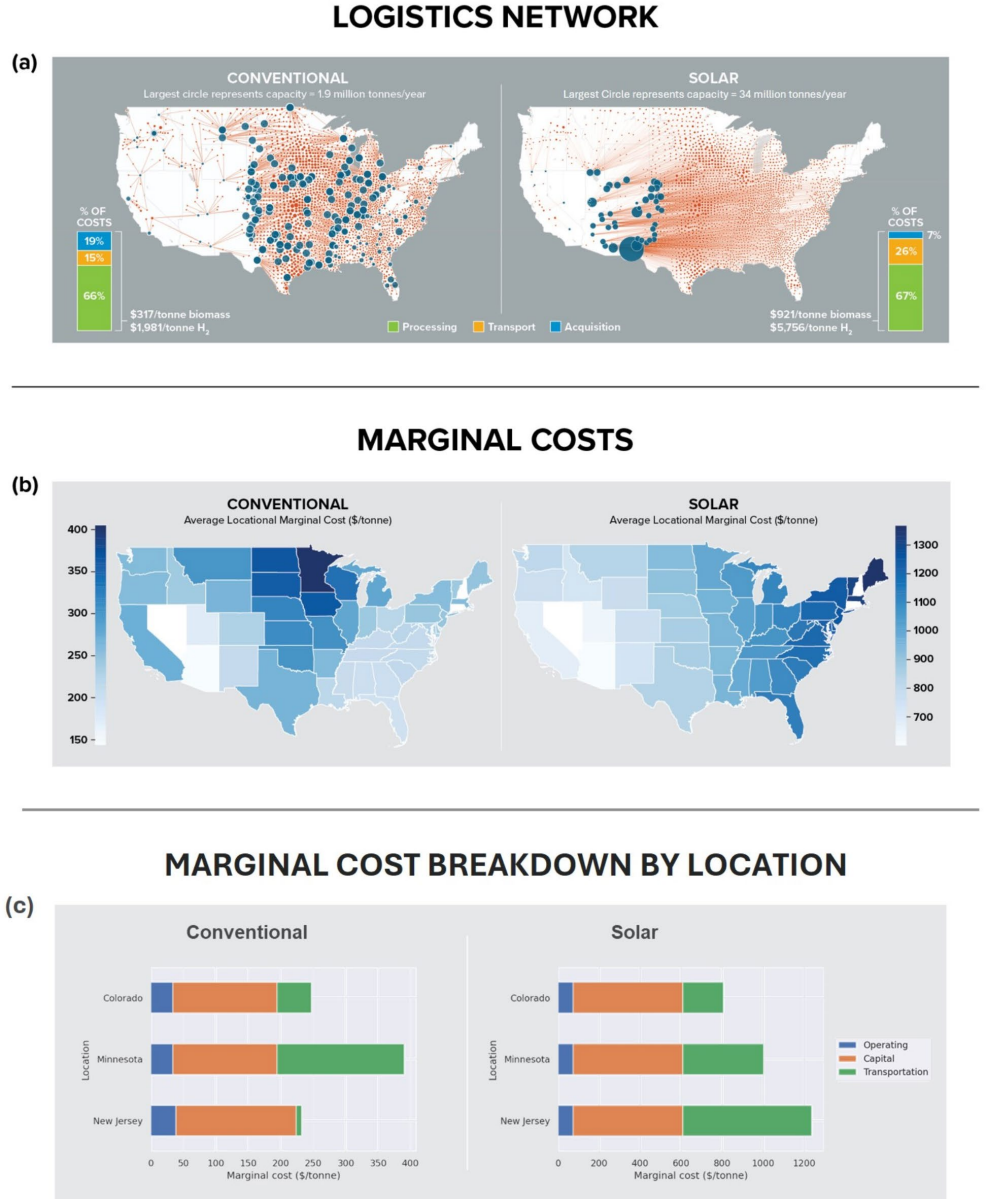
Logistics network, overall and locational marginal costs for large-scale hydrogen from biomass production in the U.S. (**a**) cost-optimized facility locations, material flows networks, and cost breakdown assuming full scale agricultural biomass utilization for SHBG and CHBG. Circles represent individual or plant-clusters scaled by capacity, and diamonds represent biomass source locations. Transportation routes between collection centers and processing facilities are shown as straight lines, but we estimate actual travel distances (see methods section). The candidate facility locations are restricted to permissible sites (see Supplemental Fig. S2). (**b**) State-level locational marginal cost for hydrogen production assuming full scale biomass utilization. (**c**) breakdown of marginal costs contributions for solar SHBG and conventional CHBG in select locations—Colorado (West), Minnesota (Midwest), and New Jersey (East Coast).

Our analysis shows that feedstock logistics is central to the economics of solar hydrogen production from biomass gasification. Figure 3 summarizes these results for the U.S., highlighting major cost contributors, as well as the relationship of cost with scale. For context, we contrast the solar hydrogen from biomass gasification (SHBG) facility with the conventional hydrogen from biomass gasification (CHBG) plant. To generate the results in Fig. 3, we made the following assumptions. First, all the biomass residue available at a baseline feedstock price of $60/tonne^8^ go to producing hydrogen. Second, the SHBG candidate locations were constrained to the southwest, which has relatively high solar intensity and follow the U.S. Bureau of Land Management’s (BLM) exclusion zone recommendations^34^ (Supplementary Fig. S1). The candidate locations for the CHBG plant have no regional restrictions but also exclude protected zones. Third, the logistics pipeline represents the baled agricultural biomass production pathway (Fig. 8), and the overall cost is made up from contributions from the solar infrastructure, the gasification plant, feedstock acquisition, and transport logistics, as detailed in the methods section. Finally, the SHBG plant operates when solar energy is available and shuts down at night.

#### Cost and network structure at scale

The cost of hydrogen from SHBG technology outstrips that from conventional CHBG due to expensive solar infrastructure and increased logistics costs stemming from resource misalignment. This is illustrated in Fig. 3a, which graphically visualizes and contrasts the logistics network infrastructure and cost for the conventional CHGB and solar SHBG technologies at scale. As shown in the Figure, CHBG facilities are located across the U.S. Their locations are determined primarily by balancing processing facility costs, proximity to biomass sources, transportation costs, as well as the relative cost of construction between regions in the U.S., captured by their respective area cost factors^35^. By contrast, the SHBG facility locations are further restricted to the high solar irradiation regions of the west coast. The consequence is that while processing costs at the facility account for roughly the same fraction (~ 65%) for both SHBG and CHBG, the specific cost of hydrogen production via SHBG is about three times that for CHBG. Two major contributors explain this difference. The first is the cost of the solar infrastructure, which triples the capital cost of the SHBG plant (Supplementary Tables S1 and S6). The second is logistics, which doubles the fractional contribution from transport costs for SHBG compared to CHBG. In absolute terms, this translates to a fivefold increase in associated transport costs at this production scale (Fig. 3a).

##### Insights from marginal costs

Marginal cost analysis shows that the cost for processing an additional tonne of biomass for hydrogen production depends on location, and the relative distribution of costs across locations depends on the deployed technology. These marginal costs are calculated by analyzing changes in the supply chain network model when there is a slight increase in demand for biomass feedstock from a specific collection area. This metric is useful for economic decision making, as facilities will only choose to process additional material if the revenue from processing it exceeds the marginal cost. In the marginal cost maps in Fig. 3b, we observe a *pyramid-shaped distribution for CHBG,* with lower values at the coasts and the peak around the Midwest. By contrast, the SHBG system’s marginal cost increases continuously as the source region moves farther away from the west coast where SBHG facilities are located to leverage higher solar irradiation. The results in Fig. 3b suggest that transportation drives locational marginal costs, and Fig. 3c—which breaks down marginal costs by contributors for Colorado (West), Minnesota (Midwest), and New Jersey (East Coast)—corroborates this observation. This result clearly suggests that interventions aimed at reducing cost of hydrogen from biomass need not be regionally uniform. For instance, SHBG hydrogen production will benefit more from interventions that reduce logistics costs in the northeast than in the west coast.

#### Reverse economics of scale

In general, scaling up technology component manufacturing towards commercial maturity or increasing the installed size of plants leads to economies of scale deriving accounting for both consolidation and production experience^36,37^. This leads to the familiar power-law scaling correlations that show specific costs reduce with plant size. Although these economies of scale generally apply to single plants, our analysis reveals a reverse economy-of-scale trend at the level of the supply chain, as increasing the scale of hydrogen production led to higher specific production costs. There are three factors that contribute to costs: the cost of biomass feedstock, the cost of deploying processing facilities to meet increased demand, and the cost of transport logistics. Biomass feedstock cost grows linearly with scale, so does not affect specific costs a scale increases (Supplementary Fig. S8). Processing facility costs also have little impact on specific costs. The reason is that at any given point, most plants already operate at capacity, so that meeting additional demand requires the installation of new plants. Therefore, at the aggregate supply chain level, what we see is effectively a numbering-up approach to facility scaling, which is also linear (Supplementary Fig. S8). We are then left with the cost of transport logistics, which, given the distributed nature of the biomass resource, generally increases super-linearly with scale. We acknowledge that there is potential for cost reductions from project construction and installation experience and address this point when we discuss pathways towards sustainable hydrogen production (see Fig. 7).

**Fig. 4:**
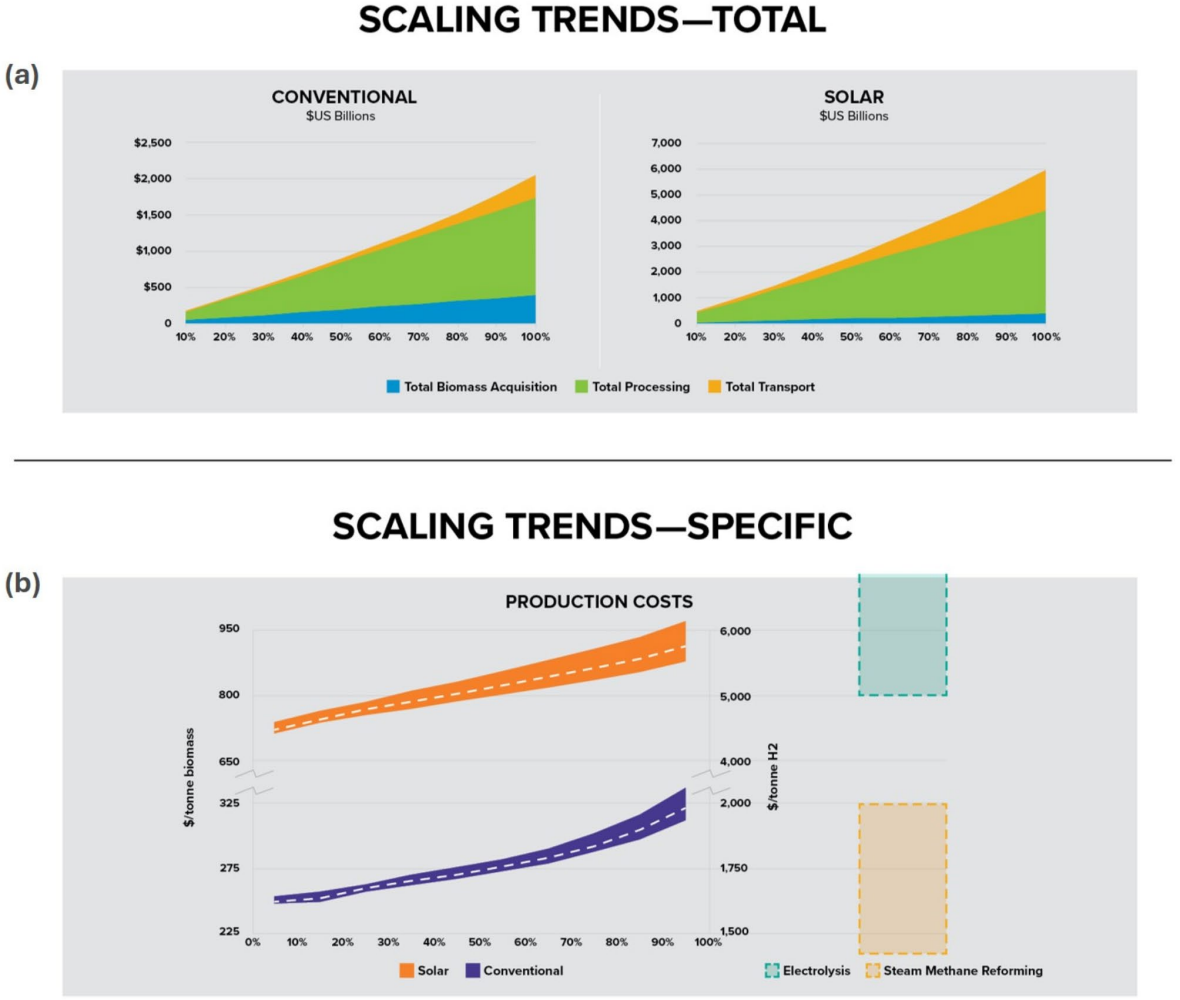
Supply chain costs and aggregate scaling trends for large-scale hydrogen from biomass production in the U.S. (**a**) Total biomass-to-hydrogen production costs as a function of fractional biomass processing scale (between 2020 and 2040), with breakdown contributions from transport, processing and feedstock acquisition. (**b**) Specific cost trends with respect to unit biomass processed, and unit hydrogen produced. Electrolysis and steam methane reforming costs are included for reference^5^.

This inverse trend observed at the level of the supply chain suggests that strategies that reduce transportation logistics costs, or that decouple logistics costs from scale of production could be as consequential to eventual technology adoption as advances in the core conversion technology. To generate this result, we evaluated multiple scenarios by sequentially increasing in the total fraction of available biomass processed from 10% all the way to 100%, which averages out to about 320 million tonnes/year from 2020 to 2040. At each scale, we find optimal locations for processing facilities and select their respective biomass sources while minimizing total costs. The results in Fig. 4a, and Supplementary Figs. S8–S9 identify the nonlinear increase in transport cost with production scale as the primary driver for this reverse trend since facilities must now source biomass from farther away locations. Transport contribution for SHBG increases from 9 to 27% (4–15% for CHBG) as biomass processing is scaled from 10 to 100%. The reverse economics of scale trend is captured in the specific cost profile in Fig. 4b. While the trend is similar for CHBG and SHBG, the magnitude is significantly different, thanks to contributions from solar infrastructure costs, and the higher absolute transport costs for the SHBG. SHBG costs compare with that for hydrogen from water electrolysis, while CHBG is closer to steam methane reforming, which currently accounts for close to 95% of the total hydrogen production in the U.S.^38^. By benchmarking against the cost of water electrolysis, this result also suggests an approximate cut-off criterion—relative to water electrolysis—for a competitive deployment scale for SHBG.

### When denser is cheaper

So far, we have shown that the misalignment between solar and biomass resources increases the contribution of logistics to SHBG hydrogen production costs. The results in the previous subsection assumed baled agricultural biomass as feedstock. Since the low mass and volumetric density of biomass feedstock drives transportation costs, alternative preprocessing strategies can change the biomass feedstock density—baled biomass has a bulk density of about 140 kg m^−3^, ground, 120 kg m^−3^ and pelletized, 600 kg m^−3^^39,40^—and their net effect on transport costs.

**Fig. 5:**
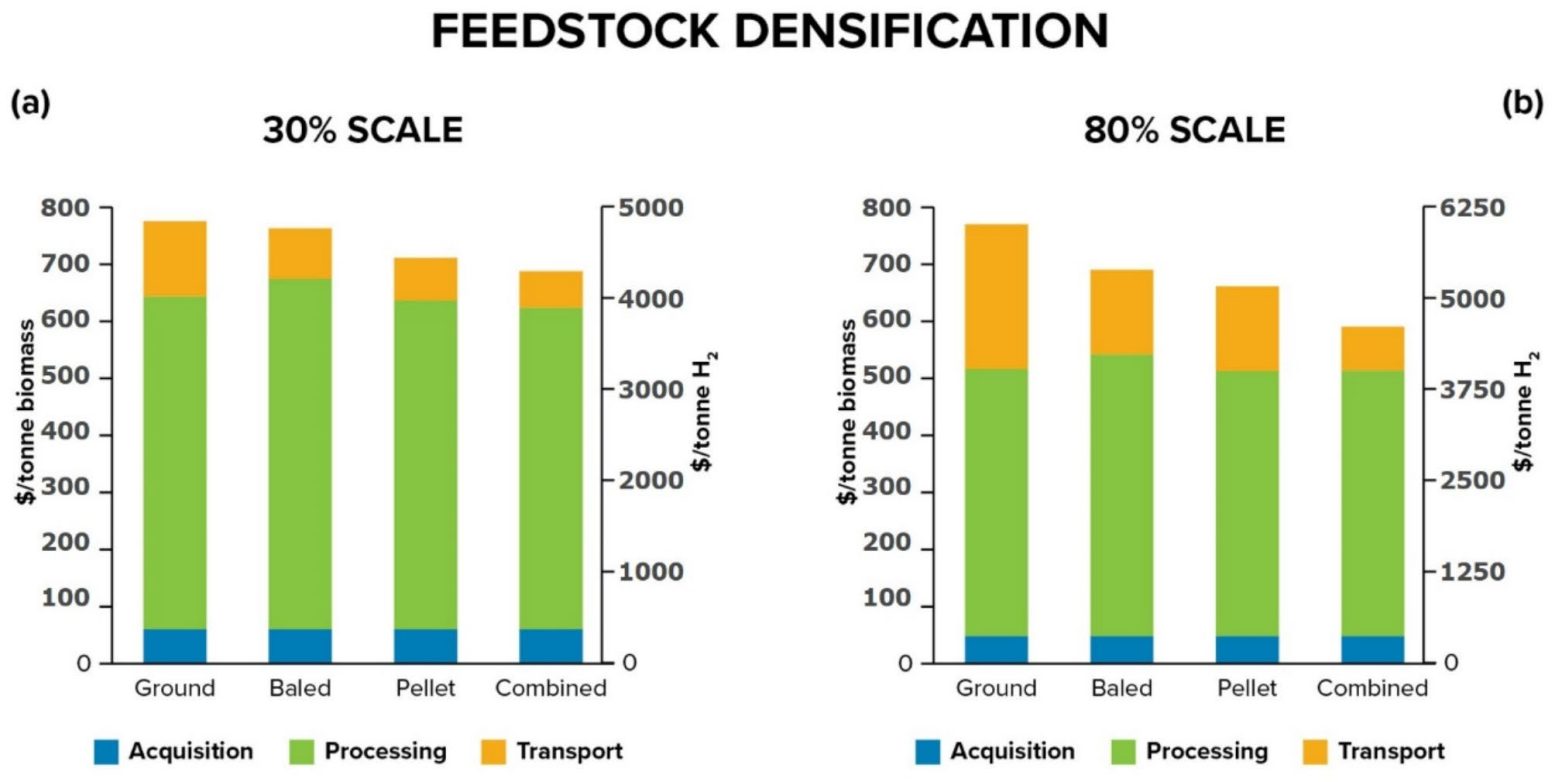
Effect of preprocessing strategy—baling, grinding, pelletizing, and combined logistics network options—on cost, with breakdown of cost contributors. (**a**) Comparing costs at 30% scale. (**b**) Comparing costs at 80% scale.

Figure 5 compares the effect of different preprocessing pathways on the cost of hydrogen production at two biomass processing scales—30% and 80%. The baled pathway represents the reference pipeline where the SHBG plant directly accepts baled biomass. The ground and pelletized pathways represent biomass to hydrogen pipelines that respectively include feedstock grinding and pelletizing before conversion in the SHBG plant. The combined pipeline finds the optimal distribution of biomass feedstock across baled, ground and pelletized pathways (Fig. 8, Supplementary Figs. S2-S5). At both scales, the ground biomass pathway proved to be the most expensive, followed by baled, pelletized, with combined providing the most economic option. Pelletizing increases density and thus reduces specific transportation cost, which brings down costs relative to alternative pathways. At 30% scale (Fig. 5a), the pelletized pipeline showed limited gains because, at this scale, the additional cost of densification counterbalances reductions in transport cost, given the relatively shorter distances travelled. Moreover, the weight limit for truck bed constrains pellet transport to utilize less than 50% of truck volumetric capacity, negating some of the potential gains from densification (see Supplementary Table S5 for details). As the processing scale increases to 80% (Fig. 5b), transportation becomes a more significant contributor to cost, and the gains from densification proportionally increase.

The combined pathway yields the most cost-effective approach since it optimally decides on a logistics strategy that minimizes overall costs by optimally splitting the biomass processing pipeline between the different pathways—in this case, about 50% is pelletized. This smart selection strategy can reduce the overall hydrogen production cost compared to individual alternatives, and the extent of realized benefits depends on the trade-off between transport and densification costs. While the logistics of biomass transport will likely grow organically with multiple, independent stakeholders, these results make the case for transportation as a service that vertically decouples biomass harvesting from processing to hydrogen, allowing the logistics entity with a more comprehensive view of the resource and facility distribution landscape to globally optimize the transportation component of the supply chain.

### Deployment under carbon penalty

Although SHBG significantly increases the cost of hydrogen production compared to CHBG, it can reduce specific emissions at the processing facility by up to 95%^22^. This raises the interesting challenge of balancing sustainability and competitiveness and requires careful consideration of the complex interplay between costs and emissions associated with both logistics and the processing technology. To this end, we explored the cost-optimal deployment of the different hydrogen production technologies under scenarios with different carbon penalties. For a more complete assessment, we also consider two other technology options: *solar hybrid* (SHHBG) and *electrified heating* (EHBG). SHHBG uses biomass combustion to provide supplemental heat when solar energy is not available^22^. This reduces capital investment for the solar plant but results in higher carbon emissions. EHBG uses electricity to heat the gasification process, resulting in specific emissions tied to average U.S. grid emissions, with the potential to evolve to lower specific emissions as the grid decarbonizes (see Supplementary Fig. S6-S7 for corresponding pipelines). However, the use of electrified heat increases operating costs (see Supplementary Table S1). We impose a carbon emission penalty for each scenario, ranging from $0/tonne and $1200/tonne of CO_2_ emitted. The goal is to understand if, and to what extent system levers such as penalties on carbon emissions could serve as a lever for incentivizing lower emission biomass-to-hydrogen technologies, with particular interest in SHBG. All simulations consider deployment at scale that uses 50% of available biomass for hydrogen production—our choice of 50% is informed by the results in Fig. 4 which suggests that at this scale, SHBG costs are just around the lower bounds for electrolysis. We consider only emissions associated with transportation, biomass combustion for energy, and emissions associated with energy for operating other plant auxiliary equipment and processes. The high-purity CO_2_ stream produced after separation from hydrogen is not considered emissions but is treated as a product whose fate—utilization or storage—is considered outside the boundary of this study.

**Fig. 6:**
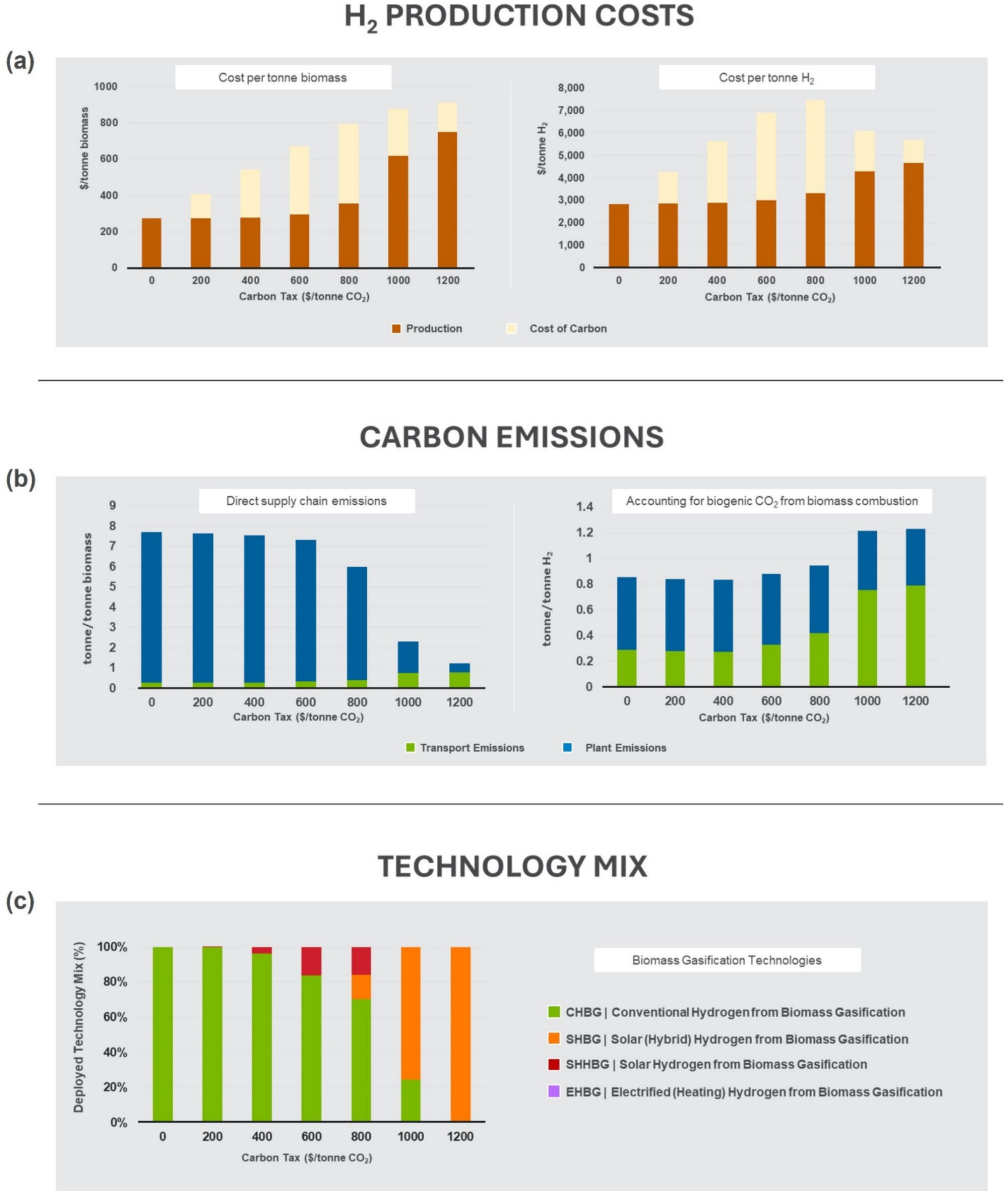
Costs, emissions and technology deployment options under carbon emissions penalty scenarios. (**a**) Effect on hydrogen production costs. (**b**) Effect on carbon emissions with and without accounting for biogenic CO_2_ from biomass. (**c**) Effect on deployed technology mix. The slightly different trends in the left and right charts in 6a arise because of differences in hydrogen yield across the different technologies.

Our analysis results indicate that very high carbon penalties are required to activate the economic deployment of solar powered hydrogen from biomass gasification technology, as summarized in Fig. 6. The stacked charts in Fig. 6a show the specific cost of hydrogen production and the associated cost of carbon emissions for different carbon emission penalty scenarios. The specific hydrogen production cost (excluding carbon penalty) stays relatively constant until a carbon emissions penalty of about $600/tonne, after which we observe sharp increases as the penalty goes towards $1200/tonne. While the specific dollar amount depends on the underlying simulation assumptions, both charts suggest that for the portfolio of technologies considered, there is not enough economic incentive to transition towards lower carbon emissions technologies at low carbon penalties. High carbon penalties will be decisive in shifting deployment towards lower emissions technologies. The left-side emissions charts in Fig. 6b show a similar but reversed trend, with a flat profile up until $600/tonne, followed by a significant drop off. Now there is an argument to be made that for plant emissions that derive primarily from biomass combustion, we have to account for biogenic carbon sequestered from the atmosphere during biomass growth. When we account for this, we obtain the chart on the right side of Fig. 6b where the overall emissions drop by an order of magnitude, transport contribution becomes a much more significant contributor across the penalty range, and the carbon penalty, which does not track biogenic emissions, becomes ineffective. In fact, if we decide to factor in biogenic carbon, there will be little incentive to go with any of the solar options and only CHBG technologies will be deployed. Currently, most carbon penalty and carbon tax mechanisms do not consider biogenic carbon, in part because of challenges with standardized accounting and allocation of emissions, and in part, because it is harder to measure and does not make for an effective incentive instrument for reducing active CO_2_ emissions.

The breakdown of contributions to total emissions in the direct supply chain emissions chart in Fig. 6b also tells an interesting story. Up to about $800/tonne carbon penalty, process emissions account for over 95% of all emissions, but subsequently falls below 30% beyond this point. The optimal deployed technology mix for different carbon penalty scenarios in Fig. 6c (Supplementary Fig. S1) explains this result. At low carbon penalties, conventional CHBG technology is favored for its economic advantages. However, as the carbon penalty rises, we see a gradual reconfiguration of the deployed technology mix with transition from a conventional technology-dominated mix to one dominated by the pricier solar technology, which offers lower emissions. The EHBG plant is not deployed within the range of conditions explored in this experiment, in part because it uses current grid-electricity which has a relatively high carbon intensity, and in part because of the high operating costs for electrical heating. However, we may expect this to change under different conditions such as lower electricity costs or a lower carbon-intensity electricity source.

### Towards sustainable hydrogen at scale

**Fig. 7:**
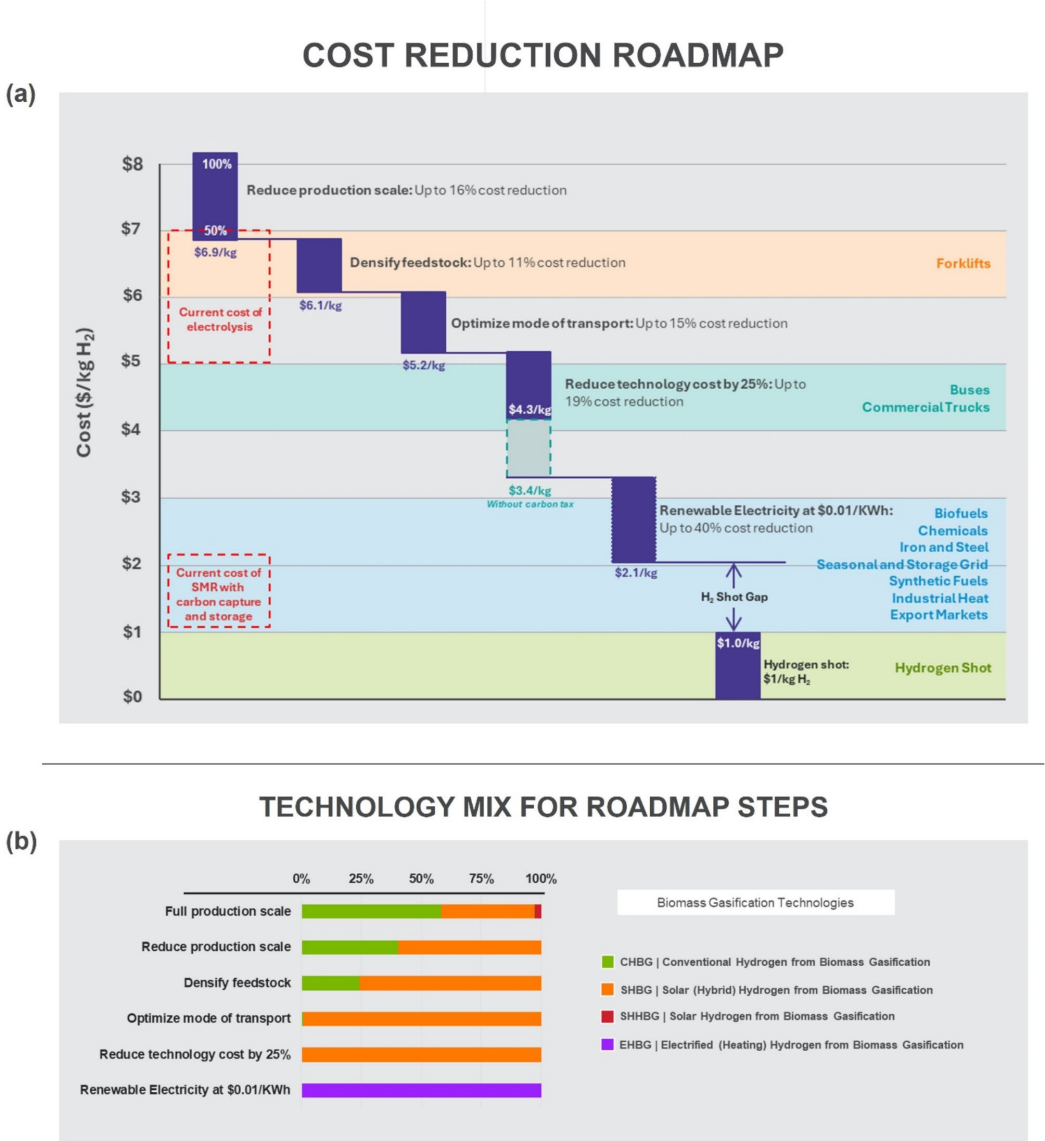
Intervention roadmap for driving costs towards the hydrogen shot. (**a**) Illustrating potential contribution of different strategies and system-level changes to reducing the cost of solar hydrogen production from biomass. The relative contributions reflect quantitative estimates from implementing each strategy, with the deficit captured as the “hydrogen shot gap”. Costs include penalties for carbon emissions, assuming a carbon penalty of $1000/tonne. The shaded regions reflect projected threshold prices that indicate the willingness of industry sectors to pay for delivered hydrogen^7^, as well as the hydrogen shot target^5^. The hydrogen shot target specifies cost at production plant gate, so does not include downstream costs associated with delivery and dispensing. (**b**) Showing the corresponding technology mix for each intervention step from simulation results.

We have shown in the preceding discussions that the typical economics of scale seen when considering a processing facility does not apply at the supply chain level with distributed biomass resources. Instead, the opposite trend is observed as the system size increases in tandem with geographic displacement of solar and biomass resources. This makes supply logistics a key driver for hydrogen production costs at scale, and consequently, an important consideration for addressing economic barriers to industry adoption. In this section, we explore the degree to which strategies and system-level changes, which directly or indirectly impact logistics, can lower hydrogen production costs. Strategy levers considered include reducing production scale, optimally densifying feedstock, adopting cheaper transportation options, achieving lower technology costs via a combination of installation experience and continuous technology refinements, and leveraging renewable electricity sources. While our specific interest is the solar-driven SHBG technology, our broader goals encompass the suite of technologies that enable sustainable hydrogen production. For this reason, we set up the study to allow the deployment of any of the technology options if favored but impose a carbon penalty of $1000/ton to disincentivize carbon emissions. Figure 7 visually illustrates the key outcomes. The results are overlayed on the threshold costs for hydrogen adoption across different industry and economy sectors in the United States^5,6^.

#### Limited production scale

**Fig. 8:**
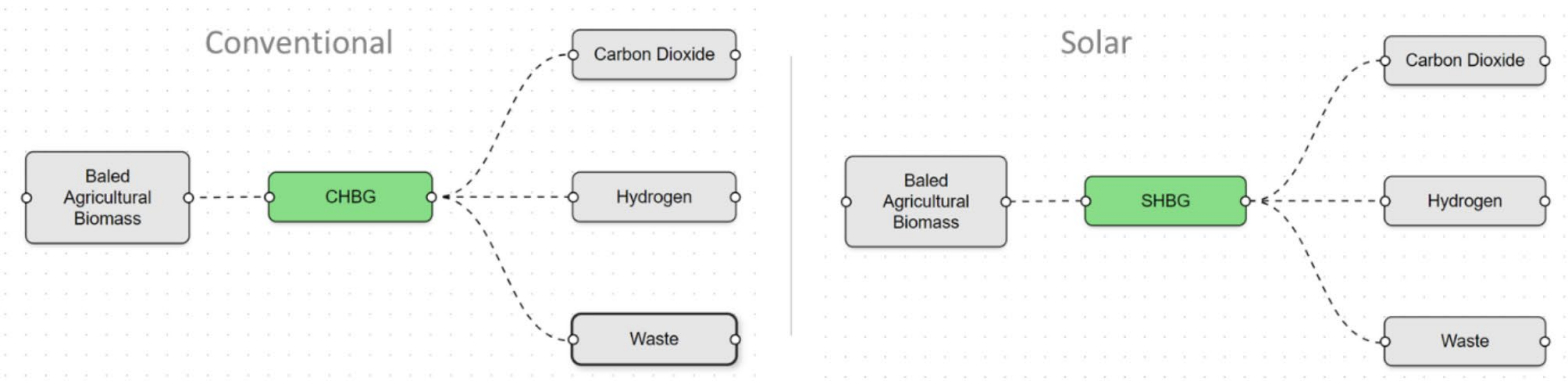
Baled Biomass supply logistics pipeline. Pipeline showing route for baled agricultural biomass with no preprocessing. The hydrogen plant is equipped with a size reduction equipment. (SHBG: solar hydrogen from biomass gasification; CHBG: conventional hydrogen from biomass gasification).

As we have shown in the preceding discussions, the cost of transportation is a major factor in the production of hydrogen from biomass, and this cost scales nonlinearly with biomass collection scale. One consideration for reducing the costs associated with transportation is to limit the scale of biomass collection. For the baled pipeline (Fig. 8, Supplementary Fig. S2), processing only 50% of available biomass residue reduces the specific cost by about 16%, where it becomes competitive with water electrolysis, and cheap enough to meet the projected cost threshold for use in heavy equipment such as forklifts (Fig. 7a). Note that this reported percent improvement as well as those mentioned for the subsequent strategy include the cost of carbon emissions, and we later show in Fig. 7 the actual cost when you remove the carbon emission costs. Also note that limiting production scale limits biomass potential match the projected hydrogen demand. Moreover, to determine a more appropriate production scale, we will need to consider competing alternative pathways for biomass residue and balance it with target production volumes and costs.

### Optimally densified feedstock

We have shown that optimized densification can reduce overall costs, and the benefits of densified feedstock increase with the required transportation distance. Allowing for optimal selection of biomass feedstock for densification (see pipeline in Supplementary Fig. S7) reduces overall costs by a further 11%. It also follows that technology advancements that reduce the cost and improve the efficiency and energy recovery of pelletization plants, or that find ways to exploit side-products from this process, can further improve overall economic outlook.

### Cheaper transport options

Switching from trucks to more cost-effective transportation modes, such as rail, could reduce specific transport costs by up to 40%^41^. Nonetheless, not all locations have access to rail lines. Furthermore, considering the competition from other high-value commodities, a hybrid approach merging the economic benefits of rail with the flexibility of short-term truck delivery can reduce costs by up to 20% less than truck transportation alone^41^. For this analysis, Fig. 7a shows that adopting lower transport options reduced costs by about 15%. This reduction comes from a balance between reduction in transport logistics costs, and the consequent shift to a mostly SHBG deployed system with higher technology costs, but lower overall carbon penalties.

### Lower technology costs

Given the massive level of deployment and capacity expansion that will be associated with scaling biomass conversion to meet projected hydrogen demand, there are a number of potential drivers that can bring costs down. These include the likely improvement in project costs from deployment experience, continuous technology and efficiency improvement for biomass handling, preprocessing and conversion, and consequent reduction in project financing risk. For this analysis, we model a scenario where these factors combine to reduce cost by up to 25%. This led to a 19% reduction in overall cost of hydrogen, with reduction in feedstock price contributing 2% and the remaining 17% from lower gasifier and solar field technology costs, since at this stage, given the order of our intervention sequence, solar-driven technologies virtually account for all hydrogen production from biomass (Fig. 7b). This reduction brings the costs to about $4.3/kg, where it has the potential to break into the market for buses and commercial trucks (Fig. 7a). When we remove the contribution from carbon tax, we see a further 20% drop in costs to just above $3/kg.

### Future low renewable electricity prices

Since resource misalignment increases transport costs, technologies that decouple solar distribution from logistics can allow modular plants to source feedstock locally. One such possibility is electrification, which can leverage existing electric transmission infrastructure to bring solar energy from high-intensity regions to the biomass-to-hydrogen facilities. From our earlier analysis (Fig. 6c), EHBG technology remains too expensive to deploy at the current electricity prices (we used $0.11/KWh) and grid carbon intensity. Here, we model an optimistic future scenario where the source of electricity is renewable, and the cost drops by 90% to 1.1 cents per KWh. Figure 7a shows that this step creates up to 40% further reduction in hydrogen costs down to just above $2/kg, where it begins to become competitive with SMR (with carbon capture). Figure 7b shows that for this scenario, EHBG outcompetes all other technology options, and hydrogen production costs becomes competitive for most industry sectors. While we specifically consider electrified heating for this study, our results suggest opportunities for alternatives—such as electrochemical biomass conversion^42^ and other hybrid options that leverage plasma or microwave to reduce required gasification temperature—and motivates further research to accelerate their development. Moreover, electrification also allows biomass to leverage other renewable energy sources such as wind.

### Bridging the H_2_ shot gap

The foregoing discussion illustrates the sequential impact of a combination of direct and indirect levers—from reducing production scale to leveraging electricity to decouple biomass and solar resource misalignment—on driving hydrogen from biomass production costs in a carbon constrained economy. The relative contributions reflect quantitative estimates from implementing each strategy, with the deficit captured as the “hydrogen shot gap”. Overall, by considering these strategies and system-level changes, we observed a reduction in costs from just over $8/kg H_2_ produced down to about $3.4/kg for a hydrogen from biomass production primarily powered by solar. When we assume the availability of cheap, renewable electricity, we see costs as low as $2.1/kg, but with a technology mix dominated by electrified heating (Fig. 7b). For SHBG, we are still faced with a gap requiring a further 70% reduction in technology costs to reach the hydrogen shot and produce hydrogen at a cost that is competitive across all industry sectors. Bridging this gap will require technology-level breakthroughs that significantly reduce the costs of solar and gasification plant infrastructure relative to current costs.

## Discussions and concluding remarks

With the U.S. biomass market projected to exceed a billion tonnes annually by 2050, biomass-to-hydrogen production can meet the projected hydrogen demand critical for decarbonizing the economy, enabling the substitution of fossil fuels with a renewable resource. Solar hydrogen from biomass gasification can further reduce carbon emissions, facilitating progress towards meeting global climate goals while improving energy resource security. However, our analysis showed that the misalignment of solar and biomass resource distribution poses a significant economic challenge, as specific production costs display a reverse economy of scale in proportion with the scale of infrastructure deployment required to process increasing fractions of available biomass residue. This reverse economy of scale is driven by increasing biomass transportation costs as biomass feedstock collection radius increases to access more of the distributed biomass, exacerbated by the misaligned distribution of solar and biomass resource. Therefore, strategies and system-level changes that reduce transportation logistics costs or decouple logistics costs from scale of production could be as consequential to eventual technology adoption as advances in the core conversion technology. We also showed that at current costs, carbon penalties are not cost-effective levers to preferentially activate the deployment of solar powered hydrogen from biomass gasification technology, and only make impact at values several times larger than in existing carbon markets^43^.

Building on these outcomes, we highlighted a series of strategies and system-level changes ranging from limiting production scale to adopting cheaper transportation. By addressing key cost drivers such as supply logistics, transportation, and technology efficiency, each of these strategies incrementally contributes to decoupling biomass supply logistics from scale of production. Together, they offer a pathway toward a more sustainable and cost-competitive hydrogen production from biomass. This not only supports decarbonization goals by providing a low-carbon alternative to conventional hydrogen production methods but also highlights the importance of integrating renewable energy sources into biomass conversion processes. Furthermore, these insights emphasize the need for continued innovation in both technology and infrastructure to overcome economic barriers, bridge the H_2_ Shot gap, and ensure that hydrogen is produced at costs that are competitive across most industry sectors.

While this study focused primarily on direct emissions from production and transport energy, it is important to consider lifecycle emissions and other socioeconomic impacts of the solar biomass-to-hydrogen production, from the upstream biomass production phase to the downstream hydrogen product distribution to end-users. Studies have shown that when not carefully managed, biomass production can negatively impact ecosystem services by altering land use, disrupting nutrient cycles and generate net emissions. We illustrated the impact of accounting for biogenic carbon when computing net emissions, but the actual impact depends on the degree to which sustainable practices were employed in the farming and harvesting phase. A full accounting of the overall life cycle impacts which also compares the relative GHG mitigation potential of different biomass to product pathways^44–48^ will need to be conducted to ensure a comprehensive assessment. This should also include an evaluation of the degree to which (solar) hydrogen from biomass gasification creates economic opportunities for local communities at every phase of the supply chain, conserves biodiversity, and avoids displacing important alternative land use activities^49^.

Moreover, biomass residues can be converted into a variety of alternative energy products, including hydrogen, electricity, biomethane, ethanol, methanol, renewable diesel, and sustainable aviation fuel^50,51^. In fact, given the projected volume of biomass production by 2050, it can be used to satisfy 100% of the sustainable aviation fuel demand in the aviation sector^9^. In addition to carbon dioxide removal^52^ and use for energy production, biomass can be converted into biochar which can be used in various application such as additives to sequester carbon in concrete^53^. The choice of which product to prioritize remains and open question, and will depend on several factors, such as the specific application, technology readiness level, underlying economic competitiveness, and ability to leverage existing supply chain infrastructure for each class of products^54^. In addition, consideration for maximizing other global objectives such as renewable energy utilization, fossil fuel displacement, and other climate benefits of biomass residue use for each product choice is equally important^50^. For example, when biomass residue is prioritized for maximizing climate benefits, producing hydrogen from biomass residue (coupled with carbon sequestration) may offer lower life cycle emissions compared to fuel production. Biomass conversion to hydrogen provides greater opportunity to capture CO_2_ during production because all carbon is gasified to produce hydrogen, while in fuel production, some of the carbon still remains in fuel and released to the atmosphere when combusted^52^.

There are additional considerations that were not included within the scope of the current analysis but would be useful to better contextualize the outcomes of this analysis. First, biomass as a resource is subject to seasonal variability, and could be vulnerable to unplanned seasonal disruptions depending on crop yield, weather, and land use changes. This variability could disrupt the supply of locally sourced biomass feedstock, necessitating acquisition from further away regions—higher transport costs—or lower plant utilization in response to the shortfall in feedstock supply. One way around this is to integrate medium-to-long term storage. But baled and ground biomass are not very amenable to long-term storage; more stable and denser preprocessed feedstock—such as pellets—would be required for longer term storage. With storage comes safety considerations, where the danger of fire could be significant worry, and fire prevention infrastructure increases the associated cost.

Second, technologies such as electrified heat for gasification or electrochemical biomass conversion^42^ can disrupt the coupling between biomass and solar resource distribution and mitigate the cost of supply logistics at scale. These technologies can become competitive at low electricity prices and in an economy with a net-zero emissions electric grid, especially if the logistics network has access to electrified or other net-zero emission transportation. However, they are either still prohibitively expensive, nascent, or not yet deployed at scale. They need further R&D to address the technical requirements—such as energy efficiency, conversion efficiency and product selectivity—and cost targets. These can be achieved via advances in areas such as understanding and tailoring electrochemically favorable reaction pathways, discovery of better catalysts for improved catalytic activity—including biomass-derived biochar^55^ –, integration of unconventional energy sources such as plasma or microwave to reduce the heating requirements, improve energy efficiency, and synergistically interact with catalysts to improve conversion efficiency. Nevertheless, even at the projected low electricity prices, these technologies will have to compete with hydrogen production from water electrolysis.

Finally, while this study focused on the upstream logistics, the downstream logistics of delivering hydrogen to the final end-users is a critical component of the hydrogen economy and would be required to complete the supply chain picture. A deeper understanding of the unique challenges and potential solutions for downstream hydrogen transport, including transport media and infrastructure, have been explored in a number of strategy reports and academic studies on the subject ^56–58^. A burgeoning hydrogen economy with wide access to transport and dispatch of the hydrogen product would favor solar hydrogen from biomass, while the production of dense chemicals fuels can simplify and reduce the cost of product-side logistics, which is equally important for technology viability. A lot will also depend on market demand for hydrogen, understanding that differences in local conditions may favor different products and hence production pathways. For instance, co-proximity to biomass resource and an iron and steel plant may favor hydrogen production. Recent studies have shown improved economic outlook for hydrogen production from biomass by improving process and material efficiency while valorizing other side products^59^. The solution may lie with developing flexible biorefineries that can tailor product distribution to prioritize short, medium and long-term demand signals from the hydrogen market. In summary, it will be important to consider all the different ways that biomass can be used, as well as the economics and environmental impacts of each use. Future work will integrate all these considerations in an end-to-end, comparative, supply chain analysis.

## Methods

### Overview

This study analyzes the logistics cost burden imposed by geospatial misalignment of solar and agricultural biomass resources, leveraging a high-level spatiotemporally resolved logistics analysis to inform outcomes. We build the analysis on the supply chain pipeline for producing hydrogen from distributed agricultural biomass resource in the U.S. We employ a spatiotemporally resolved logistics framework to assess the economic implications of solar-biomass resource misalignment and explore the impacts of production scale and preprocessing options, while accounting for environmentally informed location restrictions. We use this framework to create a cost-optimal representation of a biomass-to-hydrogen infrastructure network in continental U.S, and leverage this representation to determine the number, size and geographic locations of facilities, and associated biomass and biomass-derived flows. We implement the logistics optimization problem in RELOG^60^, an open-source software package for logistics optimization designed to model and analyze customized logistics pipelines with multiple types of plants, multiple types of products and multiple time periods. This analysis considered a 20-year window from 2020 to 2040, using the annual biomass availability and distribution data visualized in Fig. 1a. One of the key decisions underlying our modeling in this study is to locate the solar thermal facilities in the west coast of the United States, and we did this for a number of reasons. The first and most important of them is the availability of solar irradiation at the intensity and distributed scale required to support the projected hydrogen. Other reasons include the as the presence of an ecosystem of infrastructure, talent, technical support and favorable regulatory environment for such deployment. Moreover, there is already extensive body of work supported by Bureau of Land Management that has mapped out the region to identify favorable candidate locations for solar installations, based on environmental impact considerations^34^.

### Facility techno-economics

#### Model structure

The facility techno-economic model comprises three main components: the solar plant sub-model that provides the required process heat, and the biomass gasification plant sub-model that handles hydrogen production, and the pre-processing plant sub-models for grinding or pelletizing biomass. For each sub-model, the economic analysis applies a bottoms-up approach that uses reference sizing information to determine the corresponding equipment costs, capital and operating costs. For the reference biomass gasification model, we use plant characteristics and costs adapted from the DOE hydrogen production cost estimates based on the Hydrogen Production Cost Models tool developed by NREL^15,36,61^. The cost estimates excludes biomass feedstock costs, which we obtain from the Billion-Ton report^8^. Economic data for biomass preprocessing equipment—pelletizing and size reduction—were obtained from different literature sources.^8,40,62,63^ To keep things simple, we model pre-processing as a service for which we pay the equivalent variable operating cost per tonne of biomass processed. For the solar plant sub-model, we use the economic model described by Boujjat et al.^22^ which comprises a heliostat field and reflecting (beam-down) tower technology. The reference costs were estimated adding up the cost of the different components and sized to match the thermal energy demand of the biomass gasification plant. Details of all the parameters and implementation for this model is provided in the accompanying TEA workbook (Supplementary Table S6).

#### Technology options

For this analysis we modeled four technology options for biomass gasification. The first is the conventional hydrogen from biomass gasification (CHBG) plant, which represents the incumbent technology that uses biomass combustion as energy source to drive the gasification process. The second is the solar hydrogen from biomass gasification (SHBG) plant, which uses solar thermal heat to drive gasification, and shuts down when solar energy is not available. The third is the solar hybrid hydrogen from biomass gasification (SHHBG) plant, which primarily uses solar thermal heat to sustain gasification, and biomass combustion to make up for any solar deficit. The fourth is the Electrified hydrogen from biomass gasification (EHBG) plant, which converts electricity to heat the biomass gasifier. We assume that the electricity comes originally from solar, therefore this plant incorporates the resulting energy conversion inefficiencies. The CHBG plant is adapted from the NREL model^36^ and the SHBG and SHHBG models are adapted from Boujjat et al.^22,23^. For simplicity, we assume in all cases that the gasifier is indirectly heated, and that since the gasifier cost is not dominant, the adjustments to gasifier design to accommodate the different heating sources has only marginal impact on the capital cost, Therefore, the resulting differences in costs were neglected.

#### Scaling costs

We based this economic model on a reference hydrogen production capacity of about 155,000 kg/day^22^ for the conventional plant and based on that, determined the reference biomass processing capacity, which was kept the same for the other technology options. Given differences in hydrogen yield per tonne of biomass across the different technology options (Supplementary Table S3), the model adjusts accordingly for the corresponding hydrogen production capacity from each type of facility. To estimate costs at a different production capacity, we used the scaling rule described in Eq. (1). Here $C_{target}$ is the target cost item (capital, fixed) for the scaled plant, ${\text{m}}$ is the scaling factor, $C_{ref}$ is the cost item for the reference plant, and $f_{target} ,{ }F_{ref}$ refer to the target and reference plant quantities.

1 $$C_{target} = \left( {\frac{{f_{target} }}{{f_{ref} }}} \right)^{{\text{m}}} * C_{ref}$$

For this study, m is taken to be 0.78^36^ for the gasification plant unit, capturing the economy of scale typical for chemical plants. The cost/capacity plots are shown in Supplementary Fig. S10 for the different technologies. For the solar plant, m ~ 1^22^, reflecting the fact that the scaling behavior for the solar infrastructure more closely reflects a numbering-up trend.

### Biomass logistics pipelines

#### Pipeline design

In RELOG, reverse logistics pipelines are described by two main model components: (i) products and materials; and (ii) plants. In this case study, we had 9 types of products and materials: (1) baled agricultural biomass; (2) pelletized biomass; (3) biomass waste; (4) ground biomass; (5) hydrogen gas (H_2_); (6) carbon dioxide, and (7) waste. Figure 8 illustrates the baled biomass pipeline for conventional and solar hydrogen production pathways. Baled agricultural biomass is the primary source material. Pelletized biomass, and ground biomass are intermediate products (for the respective pipelines), whereas the remaining products are final products. In RELOG, plants receive a single type of material and convert it into multiple others. This study includes two types of pre-processing plants for grinding and pelletizing biomass, and the biomass gasification plants that take baled, ground or pelletized biomass and produce hydrogen. Thus, the most complex supply chain has a maximum of $N_{pipeline} \left( {1 + N_{technology} } \right) - 1$ plants, where $N_{pipeline} = 3$ is the number of logistics pipelines (baled, ground and pelletized), and $N_{technology} = 4$ is the number of processing technology options (CHBG, SHBG, SHHBG and EHBG). Simulation experiments for the different scenarios use either individual pipelines/technologies, or combinations of pipelines and technologies as appropriate. Supplementary Figures S2-S7 illustrate the different biomass supply logistics pipelines considered in this study. The first pipeline (Fig. 8, Supplementary Fig. S2) represents the base case for baled biomass. The second and third pipelines consider respectively ground and pelletized biomass feedstock (Supplementary Figs. S3–S4). In the subsequent scenarios, we allow the model to decide which combination of preprocessing strategies to use, based on overall cost signals (Supplementary Figs. S5-S7). Material balance data for the different plant types are included in the *TEA workbook and* summarized in Supplementary Tables S3 and S4.

#### Implementation

RELOG assumes that source materials (agricultural biomass, in this case study) are initially available at specific locations known as collection centers, which are described by their latitude, longitude and the amount of material available per year, in tons. All the remaining products only become available during the gasification process. Transporting products from one location to another incurs a transportation cost ($/km/tonne), spends some amount of energy (J/km/tonne) and generates emissions (tonne/tonne). To process each tonne of material, the model assumes that plants incur a variable operating cost ($/tonne), spend some amount of energy (GJ/tonne), and produce emissions (tonne/tonne). Plants also incur a fixed operating cost ($) per year regardless of the amount of material they process, as long as they are open. Plants can be built at locations within a set of specified candidates. For the solar plants, candidate locations were selected based on the U.S. Bureau of Land Management’s (BLM) exclusion zone recommendations^34^ for applying geospatial restrictions on possible solar facility sites (Supplementary Fig. S1). This excludes road, rail and water ways, as well as areas covered by BLM’s exclusion categories such as national conservation areas, critical habitats and areas of critical environmental concerns ^34^. Candidate locations for the conventional or electrified plants have no regional restrictions but equally exclude reserved and protected zones. Opening a plant (i.e., if a plant starts operating at a certain time period) incurs a one-time opening cost ($), which is region-specific. Plants also have a limited capacity (in tonnes), which indicates the maximum amount of input material they are able to process per year. For each candidate location, we specify minimum and maximum capacity of the plants that can be built at that particular location. Different plants sizes have different opening costs and fixed operating costs. After a plant is built, it can be further expanded in the following years, up to its maximum capacity. The input JSON files contain all the relevant specifications for plants and products (see Supplementary Table S6).

### Transportation estimates

#### Costs

To determine the transportation costs for the different types of biomass—baled, ground and pelletized—we need information on their respective densities, the load capacity and cargo volume of the transport trucks, and the prevailing transport costs per mile. Density data for the different biomass feedstock types ^39,64^, truck dimensions and loading capacity data, and data on transport costs per mile were obtained from the indicated literature sources. The truck loading capacity limits the maximum biomass freight tonnage, and depending on density, could be significantly below the truck’s volumetric capacity. Transportation cost is then given by trucking cost ($/km) divided by the minimum of volumetric and weight capacity of the truck. We consider uncertainty in transport costs and define upper bound of 40% above our reference estimate, and a lower bound of 25% below our reference estimate. These values were determined by running reference case simulation, determining the effective average transportation costs ($/mile), and comparing it with the representative maximum (Sorghum) and minimum (Miscanthus) transport costs for different agricultural biomass types as reported in the Billion Ton Report^8^. See Supplementary Table S5 for summary transport data, and the TEA workbook (Supplementary Table S6) for implementation details.

#### Driving distances

Driving distances were estimated by RELOG using a Machine Learning (ML) regression model based on k-nearest neighbors. The model converts Euclidean distances between two points to an approximate driving distance and was employed to reduce the computational burden of computing accurate routes and driving distances from every biomass collection center to every candidate plant location. The model was trained on 1.2 million pairs of locations in the U.S., with training labels being calculated with OpenStreetMap and Open-Source Routing Machine (OSRM). Model evaluation showed high accuracy, with an average *mean absolute percentage error* (MAPE) of only 3.0%.

### Logistics optimization

To find an optimal biomass processing infrastructure, we use RELOG^65,66^ to solve a *mixed-integer linear optimization problem* that determines: (i) where should plants be built, (ii) how large should these plants be, and how much should they be expanded; and (iii) how should the material flow across the network. This problem is a variation of the classical *facility location problem* with multiple echelons, multiple time periods, storage and plant expansion. For the current study, we reduced computational complexity by aggregating data for the 2020–2040 simulation window into an equivalent single period problem. Equations (2) to (8) present a simplified mathematical formulation for the problem. We refer to RELOG’s documentation^65^ for a more complete description. Let $L$ be the set of collection centers holding the biomass to be processed, let $M$ be the set of final products from the SHBG plants, let $P$ be the set of potential facilities to open, and let $T = \left\{ {1, \ldots , t^{max} } \right\}$ be the set of years in the planning horizon. We minimize the overall fixed, variable, transportation and storage costs:

2 $$\begin{aligned} & minimize\;\;\;\mathop \sum \limits_{ \in T} \mathop \sum \limits_{p \in P} \left[ {f_{tp}^{open} \left( {u_{pt} } \right) + f_{tp}^{fixed} \left( {x_{pt} } \right)} \right] + \mathop \sum \limits_{t \in T} \mathop \sum \limits_{p \in P} \left[ {f_{tp}^{store} \left( {z_{pt}^{store} } \right) + f_{tp}^{proc} \left( {z_{pt}^{proc} } \right)} \right] \\ & \quad + \mathop \sum \limits_{t \in T} \mathop \sum \limits_{p \in P} f_{tp}^{\exp and} \left( {w_{p1} , \ldots ,w_{pt} } \right) + \mathop \sum \limits_{t \in T} \mathop \sum \limits_{l \in L} \mathop \sum \limits_{p \in P} f_{tlp}^{tr} \left( {y_{lpt} } \right) \\ \end{aligned}$$

where $f_{tp}^{open} , f_{tp}^{fixed} , f_{tp}^{expand} ,f_{tp}^{store}$ and $f_{tp}^{proc}$ are linear functions computing, respectively: the cost of opening $p$ at year $t$; the fixed cost of keeping plant $p$ operational; the cost of expanding plant $p$, as well as the increase in fixed operating costs generated by all previous expansions; the cost of storing material; and the cost of processing material. The linear function $f_{tlp}^{tr}$ computes the cost of transporting material from collection center $l$ to plant $p$. We have six sets of linear constraints:

- All biomass must be sent to a plant:
  3 $$\mathop \sum \limits_{p \in P} y_{lpt} = m_{lt}^{{{\text{initial}}}} \quad \forall l \in L,\;\;t \in T$$
- Amount received equals amount processed plus stored:
  4 $$\mathop \sum \limits_{l \in L} y_{lpt} + z_{p,t - 1}^{{{\text{store}}}} = z_{pt}^{{{\text{proc}}}} + z_{p,t}^{{{\text{store}}}} \quad \forall p \in P,\;\;t \in T$$
- Plants have a limited processing capacity. Furthermore, if a plant is closed, it has zero processing capacity:
  5 $$z_{pt}^{{{\text{proc}}}} \le m_{p}^{\min } x_{p} + \mathop \sum \limits_{i = 1}^{t} w_{p} \quad \forall p \in P,\;\;t \in T$$
- Plants have limited storage capacity. Furthermore, all material must be processed by the end of the planning horizon:
  6 $$\begin{aligned} & z_{pt,0}^{{{\text{store}}}} \le m_{p}^{store} x_{p} \quad \forall p \in P,\;\; t \in T \\ & z_{{p,t^{\max } }}^{store} = 0\quad \forall p \in P \\ \end{aligned}$$
- Plants can only be expanded up to their maximum capacity. Furthermore, if a plant is closed, it cannot be expanded:
  7 $$\mathop \sum \limits_{i = 1}^{t} w_{p} \le m_{p}^{\max } x_{p} \quad \forall p \in P,\;\;t \in T$$
- A plant is operational at year $t$ if it was operational at time $t - 1$ or it was built at year $t$. This constraint also prevents a plant from being built multiple times.
  8 $$x_{pt} = x_{p,t - 1} + u_{pt} \quad \forall p \in P,\;\;t \in T$$

In the constraints above, $m_{lp}^{initial} , m_{p}^{min} , m_{p}^{store}$ and $m_{p}^{max}$ are constants representing, respectively: the amount of biomass available at collection center $l$ at year $t$; the minimum capacity of plant $p$, if the plant is opened; the storage capacity of plant $p$; and the maximum processing capacity of plant $p$. The binary variables $u_{pt}$ and $x_{pt}$, indicate that plant p starts operating, or stays operational in year $t$ respectively; $w_{pt}$ describes the extra capacity (amount above the minimum) added to plant $p$ during year $t$; $y_{lpt}$ describes the amount of material sent from collection center $l$ to plant $p$ during year t; $z_{pt}^{store}$ represents the amount of material in storage at plant $p$ at the end of year $t$, and $z_{pt}^{proc}$ represents the amount of material or processed by plant $p$ during year $t$. All decision variables are non-negative. In the full optimization model, we have additional decision variables to keep track of the amount of product produced by each plant, as well as the amounts disposed. Products and materials recovered by one plant may be sent to another for further processing.

### Estimating CO_2_ emissions (life cycle analysis)

The purpose of the life cycle analysis (LCA) is to estimate the energy consumption and emissions associated with each hydrogen production option considered. However, we do not capture the full life cycle emissions. The LCA system boundary includes biomass transportation, processing (where applicable), and hydrogen production (Supplementary Fig. S12). The functional unit is per tonne of hydrogen produced. We account for energy use and the emissions associated with biomass transportation, pre-processing and hydrogen production including direct CO_2_ emissions due to biomass combustion for process heat. We estimated the transportation, and gasification energy required per tonne of H_2_ produced. The gasification energy is further broken down based on the source (solar, electricity or biomass) depending on the technology. We used the Greenhouse gases, Regulated Emissions, and Energy use in Technologies^67^ R&D model to generate greenhouse gas (GHG) emission factors of each fuel/energy type including the biogenic emissions associated with biomass combustion (Supplementary Table S7). These were then integrated with energy usage value per tonne H_2_ to estimate the associated emissions. It is worth noting that the process emissions due to biomass gasification is not included in our calculation, we assume that this is captured as part of the product from the gasification process and can be utilized as a feedstock for other purposes. In addition, the energy requirement for capture and compression is not accounted for in our analysis.

### Additional data

Data for biomass resource distribution was obtained from the Billion Ton report.^8^ Data on solar irradiance was collected from the national solar radiation database.^68^ Data on excluded zones for facility siting was obtained from the Bureau of Land Management information repository^34^ (see Supplementary Fig. S1). Disposal costs are included in the plant operating costs, as per data from the DOE- hydrogen production and cost estimate studies^15,36,61^.

### Analysis resources

Data and models associated with each simulation experiment in this study are publicly available as of the date of publication and can be accessed using the links provided in the Supplementary Key Resources Table S6. This data includes (1) input data (.JSON ) files for each analysis scenario, which can also be visualized using the RELOG graphical user interface^66^; (2) corresponding output data (.CSV and .JSON) files; (3) python scripts (in Jupyter notebooks) for reproducing the study, including generating input files and visualizing key results; (4) *TEA workbook* that implements the economic model for the different types of processing facilities and transportation, including assumptions and links to reference sources; (5) formatted output data used to generate the figures in the paper; (6) Other additional or associated data, such as annual biomass demand projections and geographic distribution.

## Electronic supplementary material

Below is the link to the electronic supplementary material.

Supplementary Material 1

## Data Availability

All input data used to generate the analysis scenarios (in JSON format) as well as the results data (in CSV format) have been deposited at Zenodo (10.5281/zenodo.7643832) and are publicly available as of the date of publication. The RELOG Case Builder^69^ provides a graphical user interface for generating, viewing and/or modifying the JSON input pipeline files.

## Code availability

The source code for the reverse logistics optimization model (RELOG v0.6.0) has been deposited at Zenodo (10.5281/zenodo.7443257) and is publicly available as of the date of publication. The python scripts for generating the input files for the analysis have been deposited as well at Zenodo (10.5281/zenodo.7643832) and are publicly available as of the date of publication. A guide for using RELOG to reproduce this type of analysis can be found in the RELOG documentation^65^ and RELOG STAR Protocol^66^.
