# Supplementary material for: Assessing the deployment of solar-driven hydrogen from biomass at scale in the U.S
Source: Sci Rep. 2025 Apr 24;15:14275. doi: 10.1038/s41598-025-90290-y (PMC12022068; doi:10.1038/s41598-025-90290-y)
Supplement: Supplementary file 1 — Supplementary Material 1 [file 41598_2025_90290_MOESM1_ESM.docx]

Assessing the Deployment of solar-driven hydrogen from biomass at scale in the U.S.

Chukwunwike O. Iloeje^a*^, Sarah Runchey ^a†^, Audrey Gallier^a††^, Doris Oke^a^, Li Yu^a^, Alinson Santos Xavier^a*^

aEnergy Systems Division, Argonne National Laboratory, Lemont, IL 60439

^*^Correspondence and requests for materials should be addressed to C.O.I. at [ciloeje@anl.gov](mailto:ciloeje@anl.gov). ^†^Sarah Runchey was a U.S. Department of Energy SULI researcher at the time of contribution. ^††^Audrey Gallier was an Argonne National Laboratory research aide at the time of contribution.

Table of Contents

[Supplementary Tables 1](#_Toc123114190)

[Supplementary Figures 4](#_Toc123114191)

[Siting Considerations for Solar 5](#_Toc123114192)

[References 8](#_Toc123114193)

# Supplementary Tables

Table S1: Processing Facility Economics

| **Capacity 1 (minimum)** | | | | |
| --- | --- | --- | --- | --- |
| **Plants** | **Capacity (Tonnes^[[1]](#footnote-2)^ biomass /year)** | **Fixed Cost ($/year)** | **Variable Cost ($/tonne biomass)** | **Opening (or Capital) Cost ($)** |
| **Grinding Depot** | 212,430 | - | 17.20 | - |
| **Pelletizing Depot** | 467,346 | - | 22.29 | - |
| **CHBG Plant**  (ground \| pellet) | 1,699,440 | 22,369,667 | 35.06  (- \| 22.21) | 607,786,100 |
| **SHBG Plant**  (ground \| pellet) | 1,699,020 | 58,088,756 | 58.65  (50.89 \| 45.81) | 1,566,324,273 |
| **SHHBG Plant**  (ground \| pellet) | 16,995,278 | 30,494,450 | 39.1  (- \| 26.25) | 829,431,419 |
| **EHBG Plant**  (ground \| pellet) | 1,699,020 | 29,416,281 | 447.93  (-\| 435.08) | 907,559,394 |
| **Capacity 2 (maximum)** | | | | |
| **Plants** | **Capacity (Tonnes biomass /year)** | **Fixed Cost ($/year)** | **Variable Cost ($/tonnes)** | **Opening Cost ($)** |
| **Grinding Depot** | 4,673,460 | - | 17.20 | - |
| **Pelletizing Depot** | 4,673,460 | - | 22.29 | - |
| **CHBG Plant**  (ground \| pellets) | 37,387,680 | 176,736,265 | 35.06  (- \| 22.21) | 6,773,926,100 |
| **SHBG Plant**  (ground \| pellets) | 37,378,430 | 770,888,590 | 58.65  (50.89\|45.81) | 20,645,628,875 |
| **SHHBG Plant**  (ground \| pellets) | 37,389,607 | 306,177,365 | 39.1  (- \| 26.25) | 9,741,624,434 |
| **EHBG Plant**  (ground \| pellets) | 37,378,429 | 255,272,519 | 447.93  (-\| 435.08) | 10,114,973,456 |

Table S2: Biomass Storage Cost ($/tonne)^1^

| **Biomass** | **Value** |
| --- | --- |
| **Baled** | 5.71 |
| **Ground** | 5.71 |
| **Pellets** | 1.52 |

Table S3: Biomass Gasification Facility material balance (tonne/tonne biomass) ^2^

| **Component (tonne)** | **CHBG**  **(baled)**  **(ground \| pellet)** | **SHBG**  **(baled)**  **(ground \| pellet)** | **SHHBG**  **(baled)**  **(ground \| pellet)** | **EHBG**  **(baled)**  **(ground \| pellet)** |
| --- | --- | --- | --- | --- |
| **Biomass** | 1 | 1 | 1 | 1 |
| **Hydrogen** | 0.096  (0.103 \| 0.103) | 0.16  (0.172 \| 0.172) | 0.107  (0.115 \| 0.115) | 0.16  (0.172 \| 0.172) |
| **Carbon Dioxide** | 1.186  (1.275 \| 1.275) | 1.983  (2.133 \| 2.133) | 1.322  (1.422 \| 1.422) | 1.983  (2.133 \| 2.133) |
| **Waste** | 0.032  (0.03 \| 0.03) | 0.054  (0.05 \| 0.05) | 0.036  (0.033 \| 0.033) | 0.054  (0.05 \| 0.05) |

Table S4: Preprocessing (grinding, pelletizing) facility material balance (tonne/tonne biomass)^3^

| **Component** | **Mass Ratio** |
| --- | --- |
| Biomass | 1 |
| Ground/Pelletized material | 0.93 |
| Waste | 0.07 |

Table S5: Transportation cost

|  | **Units** | **Chopped** | **Baled** | **Ground** | **Pellets** |
| --- | --- | --- | --- | --- | --- |
| **Density**^4^ | tonne/m^3 | 0.07 | 0.14 | 0.12 | 0.6 |
| **Weight at truck volume** | tonne | 6.95 | 13.91 | 11.92 | 59.6 |
| **Max Truck Capacity**^5^ | tonne | 23.58 | 23.58 | 23.58 | 23.58 |
| **Maximum biomass load** | tonne | 6.95 | 13.91 | 11.92 | 23.58 |
| **Transport (lower)** | $/km/tonne | 0.29 | 0.15 | 0.17 | 0.09 |
| **Transport (Upper)** | $/km/tonne | 0.47 | 0.24 | 0.27 | 0.14 |

Table S6: Key Resources Table

| **Resource** | **Source** | **Identifier** |
| --- | --- | --- |
| Deposited data | | |
| All input and output data (JSON and CSV files) for the case studies | This study | ZENODO: DOI: 10.5281/zenodo.8328673 |
| TEA workbook containing economic data, estimates, references and assumptions | This study, adapted from various sources | ZENODO: DOI: 10.5281/zenodo.8328673 |
| Data for generating paper figures | This Study | ZENODO: DOI: 10.5281/zenodo.8328673 |
| Software and algorithms | | |
| RELOG – logistics optimization model for simulating each case study | Argonne National Laboratory  https://anl-ceeesa.github.io/RELOG | ZENODO: DOI:10.5281/zenodo.5131239 |
| Interactive Python notebooks for generating simulation input and visualizing outputs | This study | ZENODO: DOI: 10.5281/zenodo.8328673 |

Table S7: Emission factors used in this analysis^6^

|  | **Unit** | **Value** |
| --- | --- | --- |
| Diesel | g CO_2_/MJ | 90.3 |
| Electricity | g CO_2_/MJ | 122.1 |
| Biomass | g CO2/MJ | 101.9 |
| Biogenic CO_2_ | g CO2/MJ | 100.1 |

# Supplementary Figures


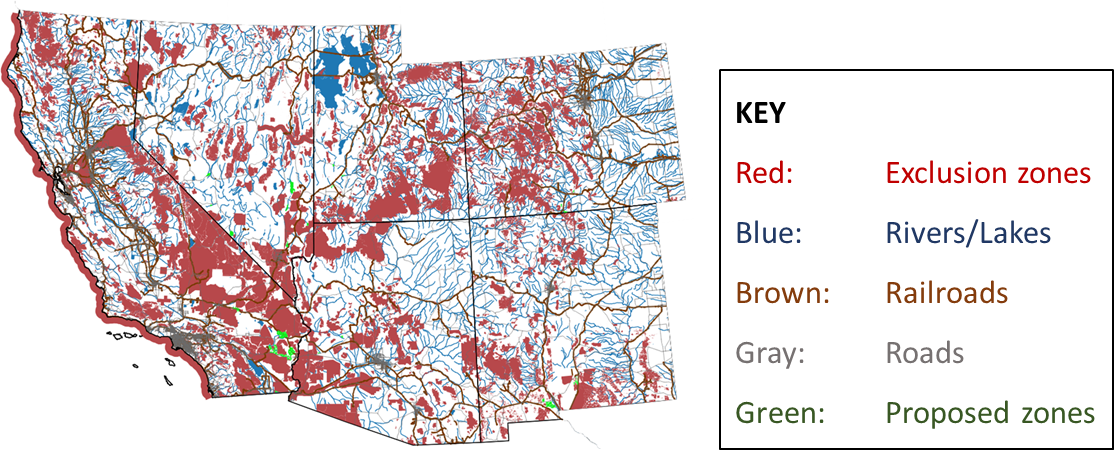


**Figure *S*1 |** U.S. Bureau of Land Management’s (BLM) *E*xclusion and Solar Energy Zone *recommendations for candidate facility locations*^7,8^*.* BLM’s exclusion zones include national conservation areas, critical habitats and areas of critical environmental concerns*. Solar Energy Zones (****proposed zones****) refer to* sites recommended as suitable for large scale solar facilities and having the minimum impact on local environment and wildlife


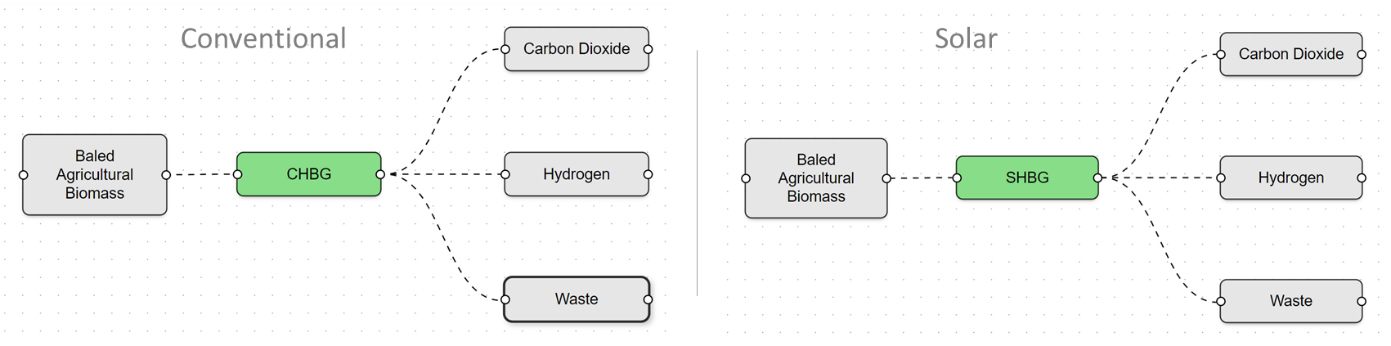


**Figure *S*2 | Baled Biomass supply logistics pipeline.** Pipeline showing route for baled agricultural biomass with no preprocessing. The hydrogen plant is equipped with a size reduction equipment. (SHBG: solar hydrogen from biomass gasification; CHBG: conventional hydrogen from biomass gasification)


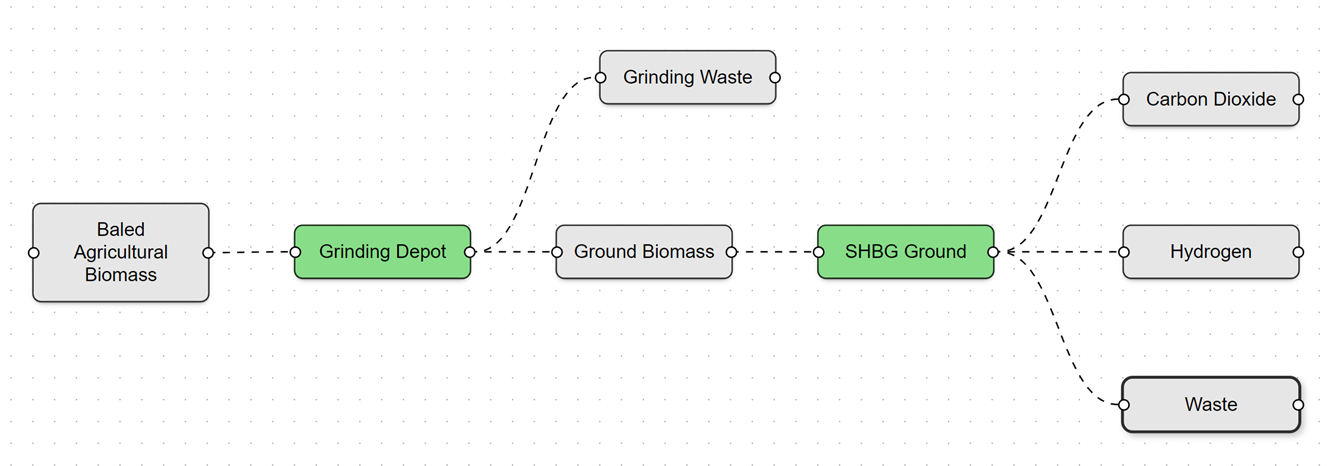


**Figure S3 | Ground Biomass supply logistics pipeline.** Pipeline showing route for agricultural biomass with grinding, where the baled feedstock is ground to the specifications of the hydrogen plant before subsequent shipping.


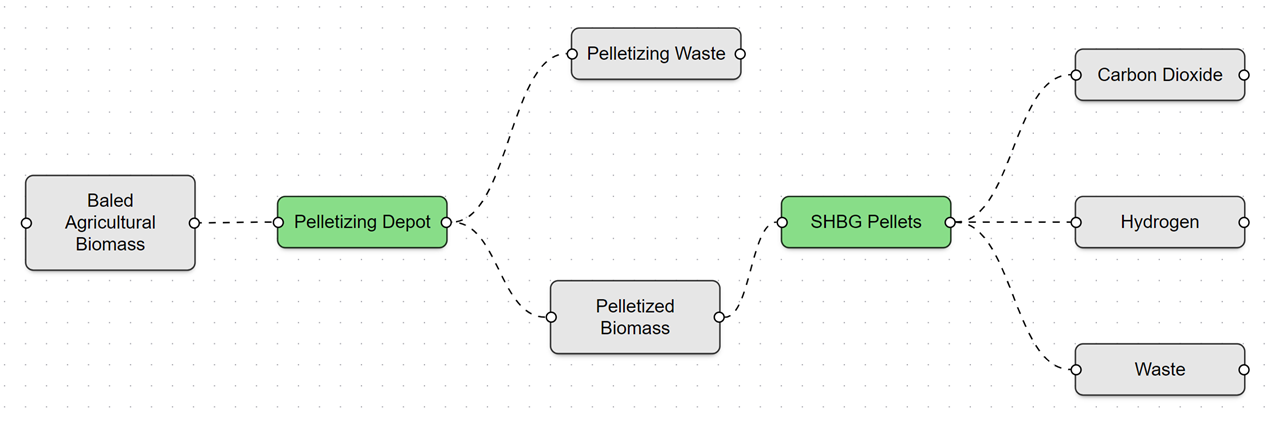


**Figure S4 | Pelletized Biomass supply logistics pipeline.** Pipeline showing route for agricultural biomass with palletization. Here the baled feedstock is sent to a densification plant where it is pelletized before shipping to the hydrogen plant


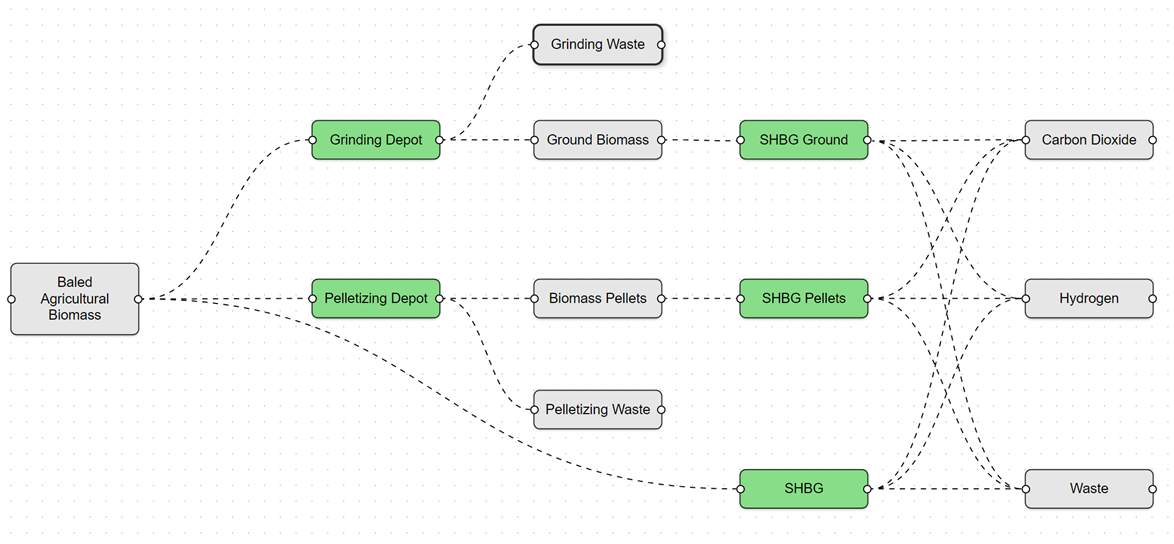


**Figure S5 | Combined Biomass supply logistics pipeline.** Pipeline including the three supply logistics routes for agricultural biomass


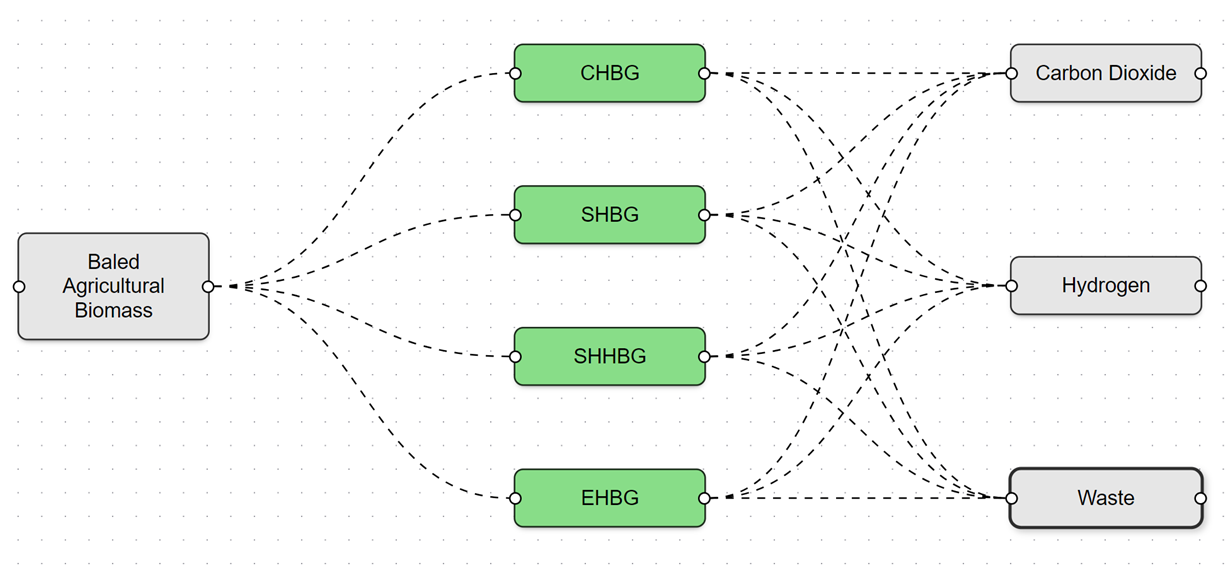


**Figure S6 | Baled Pipeline – Combined Tech supply logistics pipeline.** Pipeline for baled agricultural biomass incorporating the different processing technology options


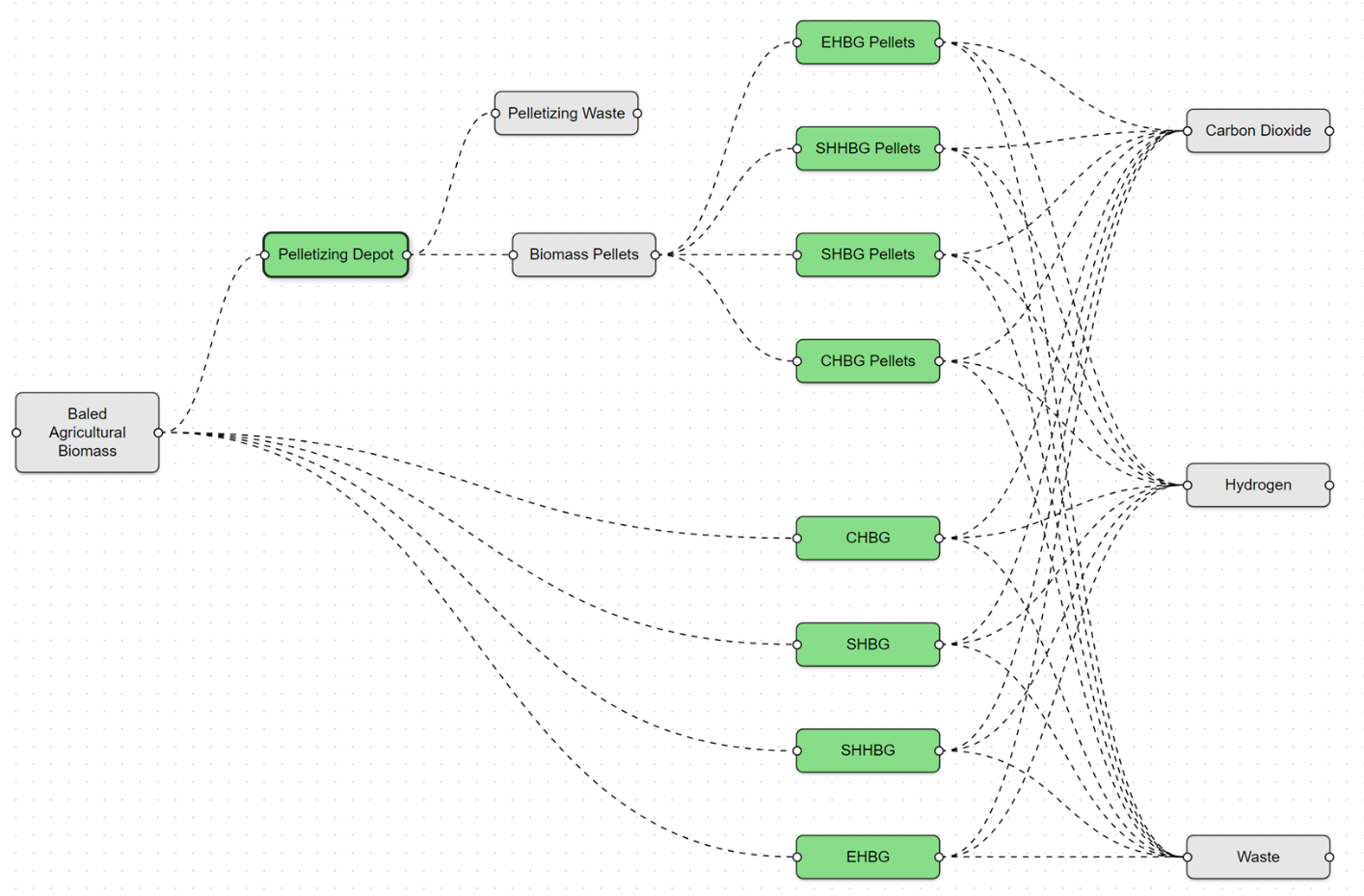


**Figure S7 | Combined Pipeline – Combined Tech supply logistics.** Pipeline including the three supply logistics routes for agricultural biomass – the ground biomass pathway is excluded from the current analysis

**
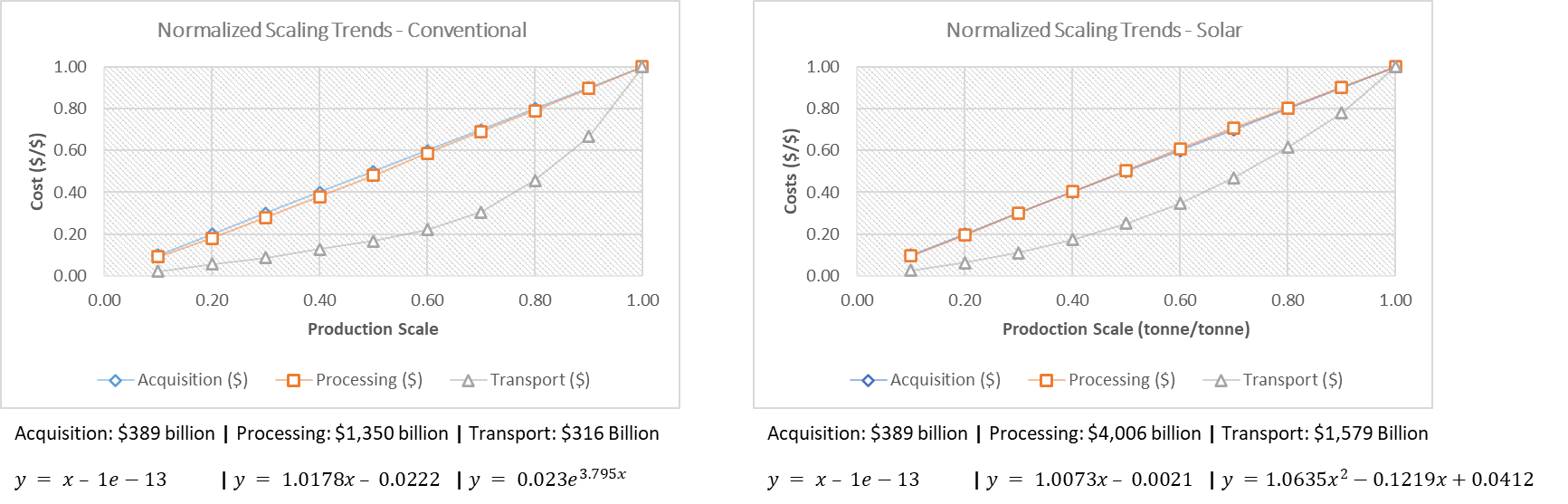
**

**Figure S8 | Normalized Total Acquisition, Transport and processing scaling plot with correlation for SHBG and CHBG**

**
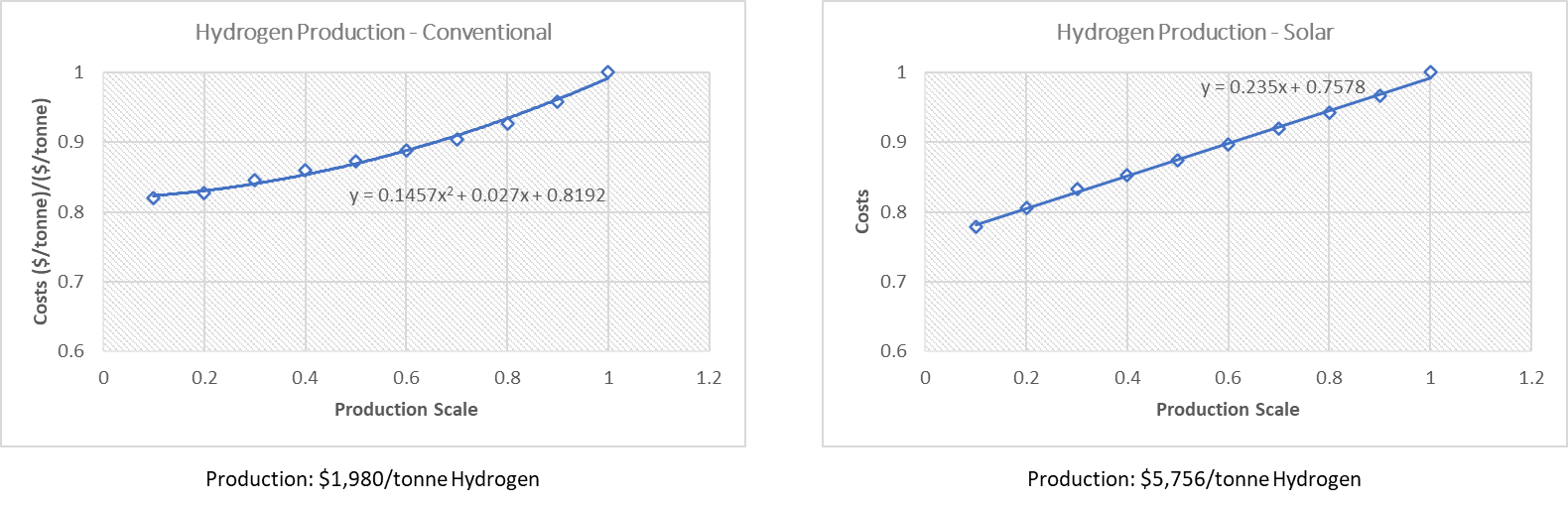
**

**Figure S9 | Specific production scaling plot with correlation for SHBG and CHBG.** Production cost includes acquisition, transport and processing costs, and does not include carbon emissions penalty

**
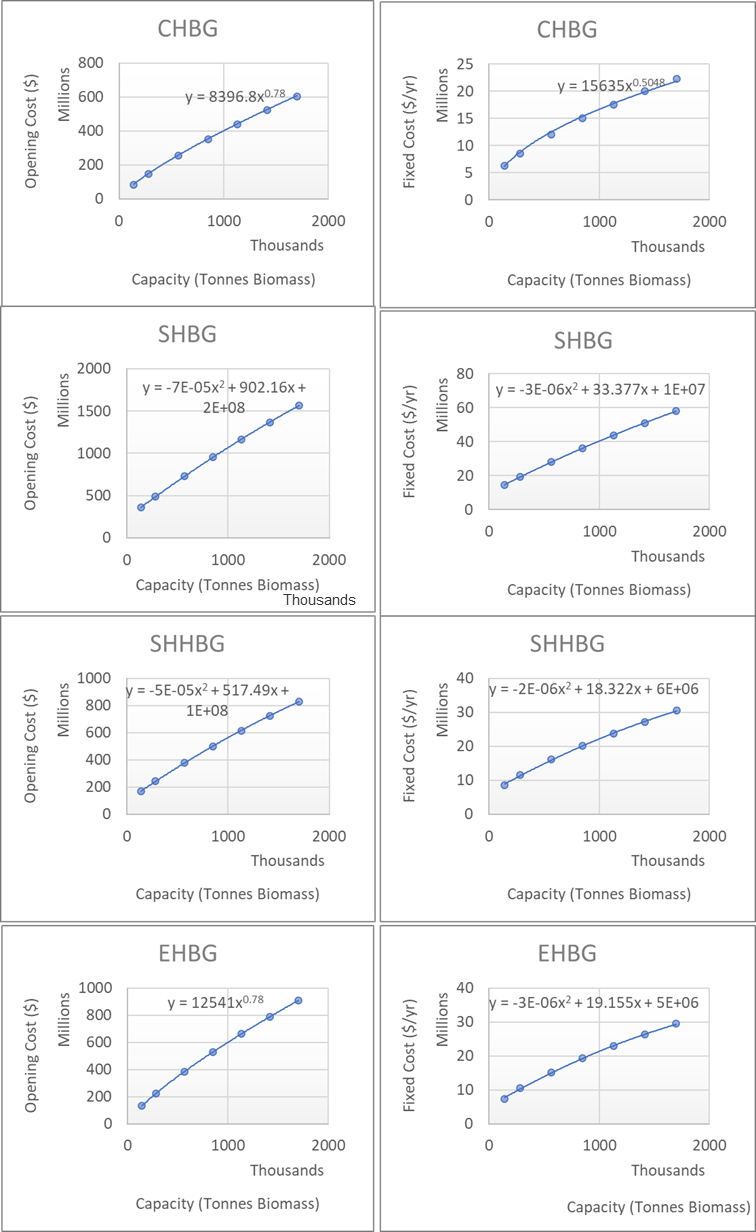
**

**Figure S10 | Facility-level scaling plot with correlation for all technology options**


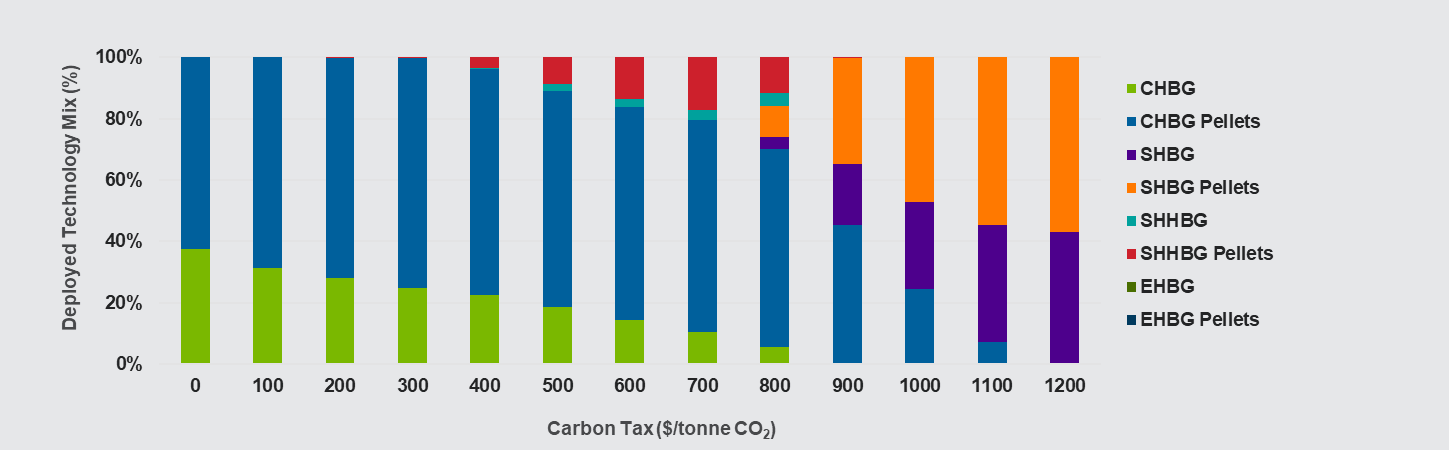


**Figure S11** | Effect of carbon penalty on deployed technology mix.


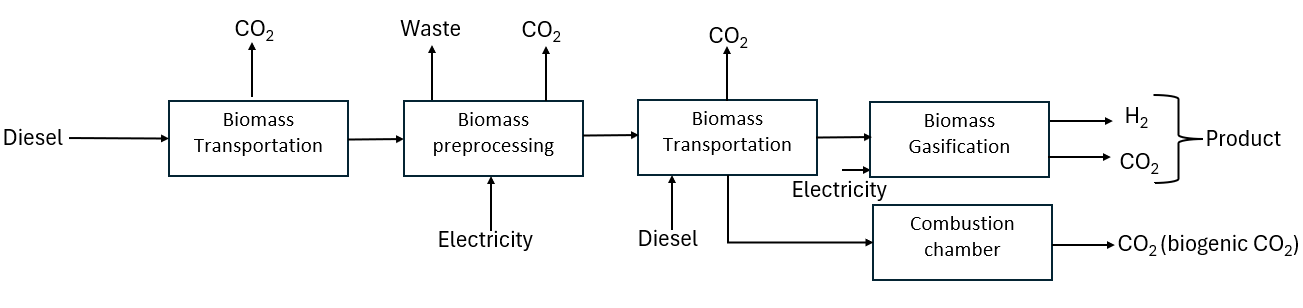


**Figure S12| Life cycle system boundary for hydrogen production used in this study**

# References

1. Sokhansanj, S., Kumar, A. & Turhollow, A. F. Development and implementation of integrated biomass supply analysis and logistics model (IBSAL). *Biomass and Bioenergy* **30**, 838–847 (2006).

2. Binder, M., Kraussler, M., Kuba, M. & Luisser, M. Hydrogen From Biomass Gasification. *IEA Bioenergy* (2018) doi:10.1016/b978-008043947-1/50003-x.

3. Langholtz, M., Stokes, B. & Eaton, L. *2016 Billion-Ton Report: Advancing Domestic Resources for a Thriving Bioeconomy, Volume 1: Economic Availability of Feedstocks*. *ORNL/TM-2016/160* vol. 12 http://energy.gov/eere/bioenergy/2016-billion-ton-report (2016).

4. Sokhansanj, S. *et al.* Large-scale production, harvest and logistics of switchgrass (Panicum virgatum L.) – current technology and envisioning a mature technology. *Biofuels, Bioproducts and Biorefining* **3**, 124–141 (2009).

5. McGuire LLC. A Guide to Truck Trailers.

6. Argonne National Laboratory. The Greenhouse Gases, Regulated Emissions, and Energy use in Technologies (GREET) Model. https://greet.anl.gov/.

7. Bureau of Land Management. Maps of Land Use Allocations, Exclusions, and Pending Applications [BLM Solar Energy Program]. *Solar Energy Program | Western Solar Plan* https://blmsolar.anl.gov/maps/exclusion-areas/ (2019).

8. Bureau of Land Management. Solar Energy Zones (SEZs) Under the BLM Solar Energy Program. *Solar Energy Program | Western Solar Plan* https://blmsolar.anl.gov/sez/ (2014).

1. *Tonne always refers to dry ton* [↑](#footnote-ref-2)
